# Peak shape clustering reveals biological insights

**DOI:** 10.1186/s12859-015-0787-6

**Published:** 2015-10-28

**Authors:** Marzia A. Cremona, Laura M. Sangalli, Simone Vantini, Gaetano I. Dellino, Pier Giuseppe Pelicci, Piercesare Secchi, Laura Riva

**Affiliations:** MOX - Dipartimento di Matematica, Politecnico di Milano, Milan, Italy; Department of Experimental Oncology, European Institute of Oncology, Milan, Italy; Dipartimento di Scienze della salute, Università degli Studi di Milano, Milan, Italy; Center for Genomic Science of IIT@SEMM, Fondazione Istituto Italiano di Tecnologia, Milan, Italy

**Keywords:** ChIP-seq, Transcription regulation, GATA-1, Peak shape

## Abstract

**Background:**

ChIP-seq experiments are widely used to detect and study DNA-protein interactions, such as transcription factor binding and chromatin modifications. However, downstream analysis of ChIP-seq data is currently restricted to the evaluation of signal intensity and the detection of enriched regions (peaks) in the genome. Other features of peak shape are almost always neglected, despite the remarkable differences shown by ChIP-seq for different proteins, as well as by distinct regions in a single experiment.

**Results:**

We hypothesize that statistically significant differences in peak shape might have a functional role and a biological meaning. Thus, we design five indices able to summarize peak shapes and we employ multivariate clustering techniques to divide peaks into groups according to both their complexity and the intensity of their coverage function. In addition, our novel analysis pipeline employs a range of statistical and bioinformatics techniques to relate the obtained peak shapes to several independent genomic datasets, including other genome-wide protein-DNA maps and gene expression experiments. To clarify the meaning of peak shape, we apply our methodology to the study of the erythroid transcription factor GATA-1 in K562 cell line and in megakaryocytes.

**Conclusions:**

Our study demonstrates that ChIP-seq profiles include information regarding the binding of other proteins beside the one used for precipitation. In particular, peak shape provides new insights into cooperative transcriptional regulation and is correlated to gene expression.

**Electronic supplementary material:**

The online version of this article (doi:10.1186/s12859-015-0787-6) contains supplementary material, which is available to authorized users.

## Background

Chromatin immunoprecipitation followed by sequencing (ChIP-seq) is a widely used technique essential to study transcription factor binding and chromatin modifications. This technique has been largely used to characterize many biological processes, enabling the creation of valuable public resources of epigenomic data (i.e. ENCODE, Roadmap Epigenomics). Due to the importance of interpreting these datasets, a large number of algorithms for downstream processing of ChIP-seq experiments have been developed [1, 2]. All these methods are usually based on the evaluation of signal intensities to detect local enrichment of uniquely aligned reads on the reference genome (we refer to them as ‘ChIP-seq peaks’). Peak shape shows high variability among the ChIP-seq experiments that investigate different proteins as well as among different genomic regions in a single ChIP-seq. This variability is not only related to peak intensity [3]. Indeed, the shapes of a transcription factor (TF) usually appear concentrated narrowly, while peaks that characterize histone marks can sometimes spread over a large region [4, 5].

Recently, peak shape properties different from signal intensity have been used in peak calling [6–8], peak ranking [9] and ChIP-seq differential analysis [10]. While the developed methods show that additional features of peak shape can improve peak detection, here we want to understand whether peak shape includes additional biological properties that have not been explored yet. Our hypothesis is that peak shape is influenced by the organization and interactions of the proteins bound to the DNA, hence we want to understand if the detection of differences in peak shape in a single ChIP-seq experiment can shed light on the binding of cooperative transcription factors. We are also interested in assessing whether the organization and interactions of these transcription factors is correlated to the genomic context and to gene expression. In order to address these questions, we propose an innovative analysis pipeline that distinguishes different shapes in a set of ChIP-seq peaks and relates the obtained profiles to several independent genomic datasets (other ChIP-seq experiments for different transcription factors and for histone marks, DNase-seq and RNA-Seq data). In our method, we use cluster analysis to evaluate whether the peaks of a ChIP-seq can be divided into groups, according to both the complexity and the intensity of the coverage function that defines them. To achieve this goal we select five shape indices, embedding the problem into the framework of multivariate statistical analysis. We also employ a wide range of statistical techniques to correlate the shape with a functional role. The software SIC-ChIP (Shape Index Clustering for ChIP-seq peaks), which computes the shape indices and clusters the peaks, is available online [11] as a command line R script.

To clarify the meaning of peak shape, we decide to study the erythroid transcription factor GATA-1 (GATA binding protein 1) in K562 cell line and in megakaryocytes. In this particular setting, we show that peak shapes contain information that can be used to shed light on cooperative binding and to identify up-regulated genes. Moreover, we apply the proposed methodology to a set of ChIP-seq experiments in K562 and we discover that peak shape can vary depending on the different binding proteins under investigation. Here we mainly concentrate our attention on the study of peak shape of transcription factors, but the same ideas can be generalized to other types of protein-DNA interactions.

## Results and discussion

### Peak shape varies among different experiments

**Fig. 1:**
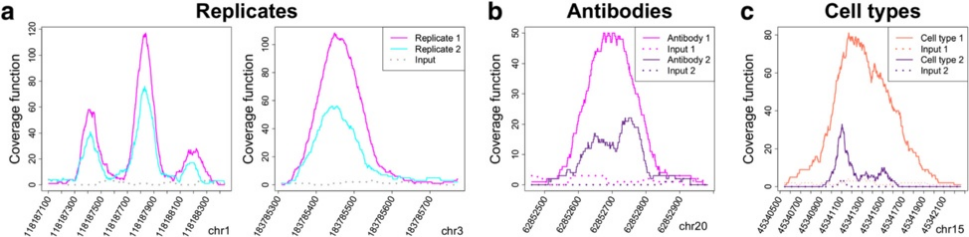
Peaks shapes.** a** Random peaks in two ChIP-seq replicates - in magenta and cyan, respectively - for the transcription factor GATA-1 in K562 cells. Despite the different efficiency of the two replicates, peak shape is highly maintained. **b** Peaks in two ChIP-seq experiments in K562 cells, performed with two different antibodies for the same transcription factor GATA-1. In this situation some peaks can show different shapes. **c** Peaks in two ChIP-seq experiments for the transcription factor GATA1 in K562 and peripheral blood-derived erythroblasts - in purple and orange, respectively. Also in this case peak shape can vary. Some relevant parameters of the ChIP-Seq plotted here can be found in Additional file 1: Table S1 and in Additional file 2

We observe that peak shape is quite reproducible as technical and biological replicates obtained with the same library preparation protocols (see Additional file 1: Table S1 and Additional file 2) give rise to the same signal in the same genomic region (Fig. 1a). This is true even if ChIP-seq efficiencies for independent experiments can vary [12] as in Fig. 1a. In addition, if two antibodies are used to perform chromatin immunoprecipitation for the same transcription factor, a subset of peaks might have different peak shapes (Fig. 1b). The antibodies may recognize different epitopes of the same transcription factor and this fact can lead to differences in shapes. Moreover, transcription factor interactions can be cell-type specific, and we observe that ChIP-seq peaks obtained using the same antibody in different cell types can show a subset of diverse shapes (Fig. 1c). These observations suggest that the analysis of peak shape may reveal insights regarding cooperation and association of transcription factors.

It is important to point out that different library preparation protocols might affect peak shape. While the read length of a ChIP-seq experiment does not have any effect on peak shape, fragment length influences peak shape as differences in fragment lengths result in different signal resolutions (larger fragments generate smoother, less resolved and bigger peak). However, the methodology we propose is not affected by differences in library preparation and sequencing, since it considers a single ChIP-Seq at a time and clusters peaks belonging to the same experiment.

### Overview of the analysis pipeline proposed

**Fig. 2:**
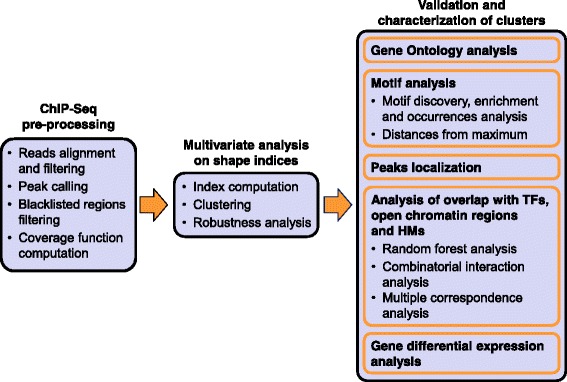
A schematic overview of the analysis pipeline proposed. After a ChIP-seq pre-processing, that involves the calculation of the coverage function for each peak found by the peak caller, multivariate clustering on five indices of intensity and shape is employed to find groups of similar peaks. Afterwards, the characterization of these clusters is studied by using GO analysis and motif analysis. Moreover, clusters are related with the presence of other proteins and with gene expression

**Fig. 3:**
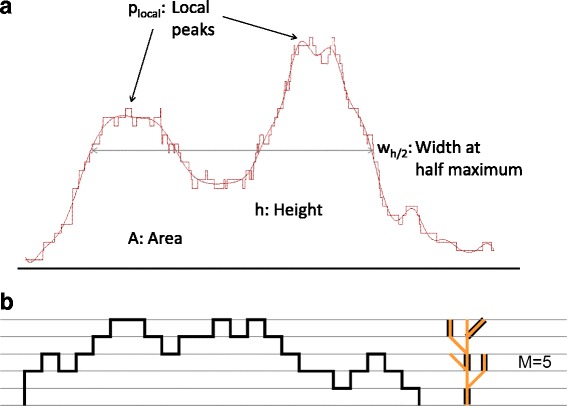
Shape indices. **a** Schema of the first four shape indices. **b** A peak and its corresponding tree, constructed as suggested in [35] and in [6]; the highlighted edges represent a maximal matching for the tree, that defines the index M

The analysis pipeline that we propose is summarized in Fig. 2. First, we perform a pre-processing step to produce coverage function and to identify enriched peaks. In this first step, we also estimate the average size of the DNA fragments obtained during sonication. We then use this estimate to extend each tag in order to get the original fragments and to compute the coverage function, counting the number of fragments that fall over each nucleotide. The correct estimation of the fragment length is essential since, as we have previously observed, peak shape can vary based on this estimation. Next, we calculate five indices of shape: the *maximum height*, the *area*, the *full width at half maximum*, the *number of local peaks*, and the *shape index M* divided by the maximum height (Fig. 3). Afterwards, we cluster peaks in the space of these resulting shape indices. We name this central part of our method *Shape Index Clustering* [11]. Finally, the obtained clusters are validated and characterized using four steps: 1) we perform Gene Ontology analysis and motif analysis; 2) we investigate the genomic locations of the peaks; 3) we study the overlap of the peaks in each cluster with peaks of other available transcription factors and histone modifications, as well as with open chromatin regions; 4) we evaluate gene expression changes in association with the shape clustering. A detailed description of each step in the pipeline proposed is given in Methods.

### GATA-1 in K562 cells

We apply the proposed analysis pipeline to ChIP-seqs for the erythroid transcription factor GATA-1 in human erythroleukemic K562 cells. The purpose of this study is to assess whether GATA-1 peak shape is associated with specific regulatory complexes and functions. GATA-1 is a transcription factor essential for erythroid and megakaryocytic development, and mutations in GATA-1 are associated with a form of leukemia found in newborns affected by Down syndrome. We select K562 cells because GATA-1 binding has been extensively characterized in this cell line [13–15] and also because K562 cell line has been widely described by several Next Generation Sequencing experiments from the Encyclopedia of DNA Elements (ENCODE) Consortium [16, 17] and from many independent investigators.

### Two ChIP-seq replicates for GATA-1 in K562 human cells

The experiments under consideration consist of two ChIP-seq replicates for GATA-1 from ENCODE [16] (GEO Accession number GSM1003608, antibody used: sc-266, Santa Cruz Biotech). The signal from a normal Mouse IgG ChIP-seq (GEO Accession number GSM935631) is used as control for peak calling. Peaks are called using MACS [18]. While the number of reads after filtering is comparable and the estimated fragment length is exactly the same in the two replicates, the number of identified peaks is different: we identify 13159 peaks in Replicate 1 and 5509 peaks in Replicate 2, with 5334 overlapping peaks (Additional file 1: Table S1). Almost all the regions selected in Replicate 2 are enriched in Replicate 1, meaning that the second replicate is much less efficient than the first one [12]. In Fig. 1a, we show the coverage function of two random overlapping peaks - in cyan and magenta for the two replicates, respectively. Despite the different degree of efficiency of the two replicates, pairs of peaks exhibit the same shape. Notably, the whole coverage function has a similar shape in the two replicates, carrying a correlation of ~0.77 on the entire genome and a correlation of 0.95 on the common peaks (Additional file 1: Figure S1).

### Clustering of shape indices leads to three clusters

**Fig. 4:**
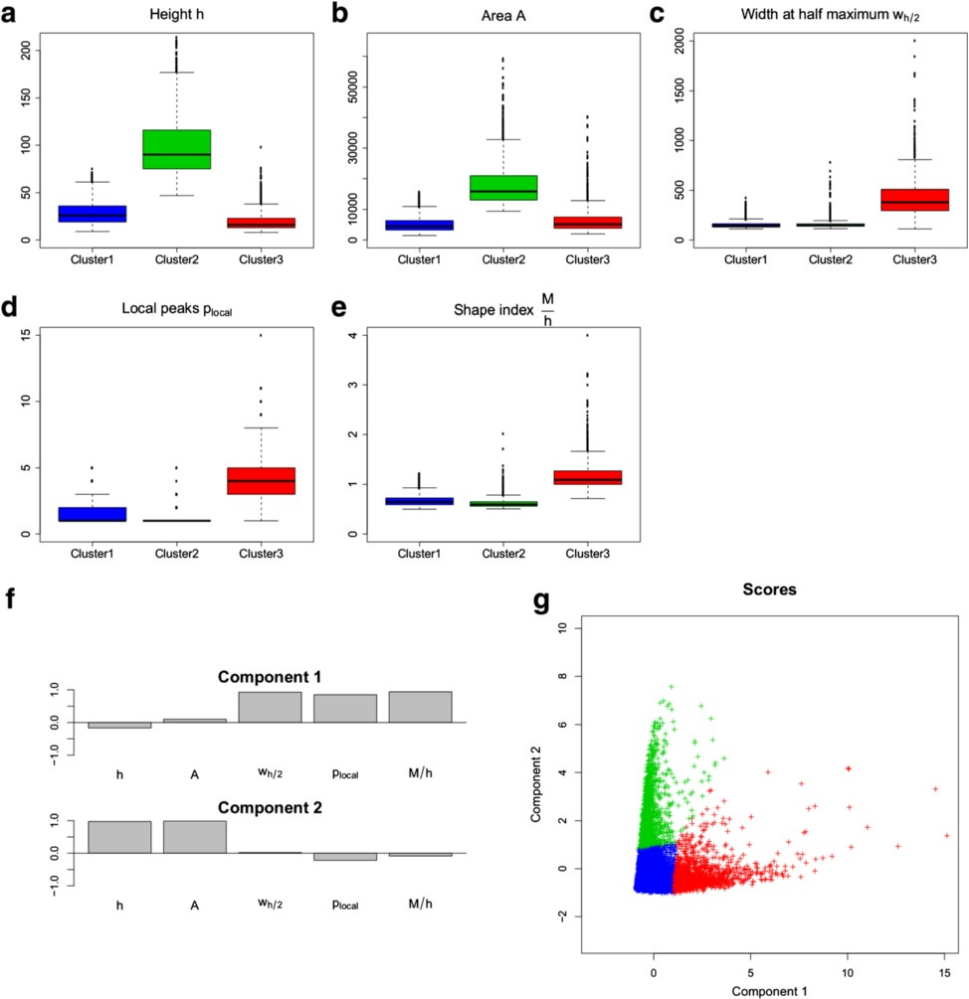
Distribution of shape indices in the three clusters. Results of *k*-mean algorithm with Euclidean distance on the standardized shape indices, in Replicate 1 for K562 cells. **a**-**e** Boxplots of shape indices in the three clusters. **f** The first two components obtained with independent component analysis, in term of the initial shape indices. **g** Scatterplot of the data in the plane defined by the first two independent components, with each point representing a peak and colored according to the cluster it belongs to

We use the statistical analysis described in Methods to assess whether there are groups of peaks inside a single ChIP-seq that can be separated according to the shape, as summarized by the five selected indices. Here, we present the results obtained by running the analysis on Replicate 1. Results concerning Replicate 2 are highly similar, despite the remarkable differences of the two ChIP-seqs, and can be found in Additional file 1: Figures S4-S6. Notably, if we merge the reads of the two replicates and then we perform the analysis, we obtain highly similar results too. From the scatterplot of the shape indices (Additional file 1: Figure S2b), from principal component analysis (Additional file 1: Figure S3) and independent component analysis (Fig. 4f-g), it is clear that the five indices are not mutually independent. Actually, the two indices related to the intensity of the signal, namely the *maximum height* and the *area* are highly correlated (correlation coefficient of 0.92); the same applies to the three indices associated with peak complexity, i.e. the *full width at half maximum*, the *number of local peaks* and the *shape index M* with correlation coefficients of 0.68, 0.74 and 0.84, respectively. However, we choose not to reduce the dimensionality of the problem in order to keep all the shape variability that we can catch with the selected indices.

**Fig. 5:**
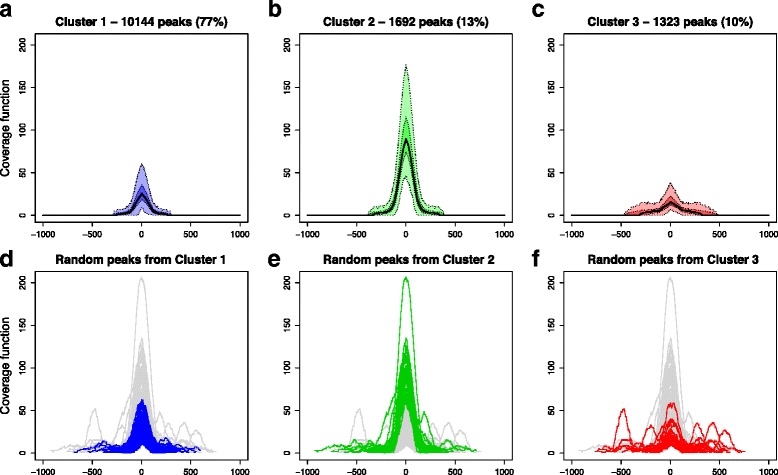
The three clusters obtained on shape indices. Results of *k*-mean algorithm with Euclidean distance on the standardized shape indices, in Replicate 1 for K562 cells. **a**-**c** Pointwise boxplots of the coverage function in the three clusters. For each abscissa, *black* indicates the median value, *dark colors* highlight the central 50 % of the distribution, while *light colors* correspond to the boxplot whiskers. **d**-**f** A random sample of 200 peaks (for visualization reason not all peaks are plotted), with colors highlighting the cluster membership. In both images, peaks are registered using as landmark the location of their maximum height

Running the k-mean algorithm on the standardized indices for several numbers of clusters *k*, we obtain the total within-clusters sum of squares plot of Additional file 1: Figure S2a: *k* = 3 seems a sensible trade-off, since the choice of a higher k is not paid off by a significant gain in the total within-clusters sum of squares. This choice leads to identify a big cluster, that comprises ~75 % of peaks (Cluster 1), and two smaller clusters including ~15 % (Cluster 2) and ~10 % (Cluster 3) of the data, respectively. According to the scatterplot of Additional file 1: Figure S2b, wherein colors indicate cluster membership, and to the boxplots of Fig. 4a-e, that display the distribution of the indices in the three clusters, Cluster 1 and Cluster 2 differ in the intensity of the peaks they contains, while Cluster 3 includes the most complex peaks. Similarly, independent component analysis (Fig. 4f-g) shows that the three clusters are well divided in the plane defined by the first two components. In particular, the component that corresponds to peak intensity (component 2) separates Cluster 2 from the others, while the component related to the complexity of the peaks (component 1) distinguishes Cluster 3. In order to better understand the shapes selected through clustering, Fig. 5 displays the pointwise boxplots of the peak coverage function in the different clusters (top panels) and the plot of a random sample of 200 peaks (bottom panels, for visualization reasons we do not draw all the peaks simultaneously). Cluster 1 is mainly composed of unimodal and not very high peaks, while Cluster 2 comprises high, bell-shaped peaks; multimodal and wider peaks belong to Cluster 3. Interestingly, peaks of Cluster 3 are not those selected with low score (less enriched peaks) by MACS: if we further reduce the threshold in peak detection, we keep on picking a considerable subset of these peaks. Hence, the groups of peaks obtained by our clustering of shape indices cannot be deduced by MACS output.

The comparison of the resulting classification for the two replicates, done on the common peaks, supports the robustness of the considered method. Indeed, nearly 90 % of peak pairs fall in the correspondent cluster of the two replicates. Moreover, only 18 pairs are misclassified between the two extreme shapes, namely between Cluster 2 and Cluster 3. In Additional file 1: Figure S7, we show the correspondences between cluster memberships of peaks in the two replicates. This cross-replicate robustness analysis and the relationship between the two replicates indicate that it is sufficient to consider the most efficient replicate for the evaluation and characterization of the clusters. Thus, in the following analyses, we show only results concerning Replicate 1.

### Only Cluster 2 is directly associated with the typical biological processes of GATA-1

We use the genomic regions enrichment of annotations tool (GREAT) to perform Gene Ontology (GO) analysis of the three clusters. GO analysis reveals that the terms related to the typical biological processes of GATA-1 (such as erythrocyte differentiation, erythrocyte homeostasis and myeloid cell differentiation) are enriched exclusively in Cluster 2 (Additional file 1: Table S2; see Additional file 1: Table S3 for the entire list of significantly enriched GO Biological Process terms in the different groups, and Additional file 3 for the complete list of terms). Considering the complete list of terms given by GREAT, it is evident that Cluster 2 is made up of few genes and many of these genes are key hematopoietic transcription factors: GATA1, FOG, and TAL1. On the other hand, Cluster 1 also contains genes that are typically regulated by GATA-1 (i.e. GATA1, GATA-2, FOG, and RUNX1), but contains also other genes associated with secondary functions of GATA-1. Indeed we can identify many genes (e.g. BAX, BAD, CASP10, BCL10, MADD, LTA, BMF) that are related to apoptosis. Interestingly, these genes are nearly absent in Cluster 2 and present at a lower extent in Cluster 3 (e.g. BAX, CASP9, BCL2L1). GATA-1 is known to inhibit apoptosis while promoting differentiation in erythroid and megakaryocytic cells [19, 20].

### Peaks of Cluster 3 contain less GATA-1 motifs

**Fig. 6:**
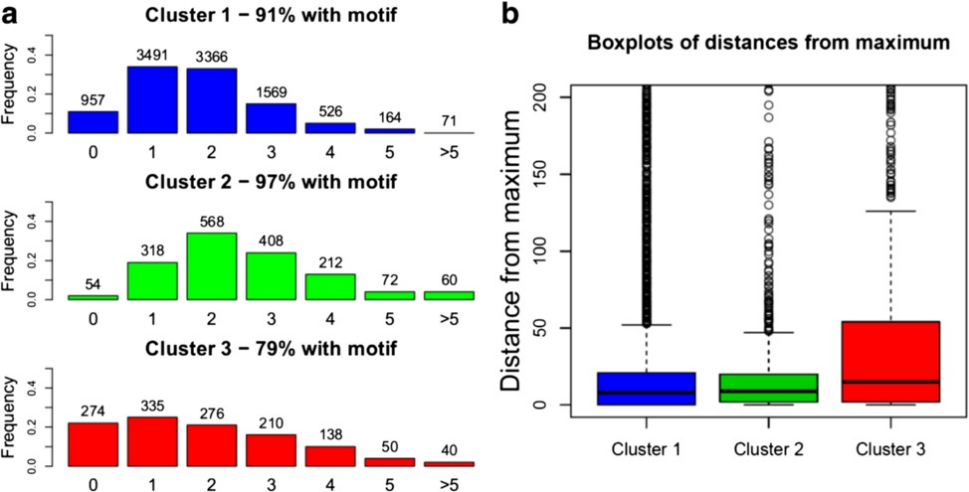
Occurrences analysis for GATA-1 motif, in Replicate 1 for K562 cells. **a** Distribution of the number of occurrences of the consensus sequence [AT]GATAA under each GATA-1 peak: under many peaks of Cluster 2 the motif occurs multiple times. **b** Boxplots of motif distances from peak maxima in the three clusters; the mean distance in Cluster 3 peaks is significantly greater than the mean distances in Cluster 1 and Cluster 2 regions (test *p*-values ~0)

GATA-1 has been shown to recognize the consensus sequence [AT]GATAA [21, 22], so we might anticipate to find this binding motif under the vast majority of peaks, whatever cluster they belong to. This expectation is only partly fulfilled. Although GATA-1 motif is found in all cases, the significance of the enrichment is different in the three clusters (see Table 1 and Additional file 1: Table S4). Surprisingly, E-values obtained with Cluster 1 and 2 are comparable to the global one. On the contrary, GATA-1 motif is less present in the peaks of Cluster 3. Only 79 % of regions belonging to Cluster 3 contain the consensus sequence [AT]GATAA, while the motif is found in almost all peaks of Cluster 2 and in 91 % of Cluster 1 peaks (see Table 1). Furthermore, peaks in Cluster 2 tend to be associated with multiple GATA-1 motifs (see Fig. 6a). Since other members of the GATA family zinc finger proteins, namely GATA-2 and GATA-3, can bind with high affinity the same motif of GATA-1 [23], the presence of more than one motif under a peak can indicate both a multiple GATA-1 binding and the simultaneous presence of several transcription factors of the GATA family. In Fig. 6b, we show the distribution of motif distance from the peak maximum. In addition of being less associated with GATA-1 motif, Cluster 3 regions exhibit a higher distance of the found motifs from peak maxima: when the motif is present, it is usually not centered near the maximum.

**Table 1:** GATA-1 motif analysis

	E-value	Peaks with motif
Global	3e-216 (3e-158)	90 % (11874)
Cluster 1	7e-258 (3e-159)	91 % (9187)
Cluster 2	2e-250 (1e-194)	97 % (1638)
Cluster 3	7e-21 (2e-88)	79 % (1049)

GATA-1 motif enrichment and occurrences analysis in the complete set of peaks as well as in the three clusters, in Replicate 1 for K562 cells. For the enrichment analysis, MEME-ChIP with default options is run on samples of 1323 peaks (the size of the smallest cluster) to get comparable E-values. In the global set and in Cluster 1, that are large with respect to the sample size, the median of E-values obtained by random sampling 10 times is reported. In parenthesis we indicate the E-values obtained running MEME-ChIP changing default parameters to allow the motif search in the whole peak regions (without trimming them). The number of peaks with the motif (occurrences analysis) is computed on the whole sets of peaks

Apart from GATA-1 consensus sequence, many other motifs are enriched in the three clusters (see Additional file 1: Table S4). Interestingly, these additional motifs are peculiar to the different clusters, suggesting distinctive types of gene regulation. All the three clusters are enriched for Ets motifs, including PU.1, GABPA, and FLI1 motifs, in accordance with what is shown by previous data on GATA-1 [24, 25]. Cluster 1 and 2, in contrast to Cluster 3, are enriched for TAL-1 and KLF1 motifs. Interestingly, TAL1 motif is usually enriched at GATA-1 activated genes [26]. In addition, Cluster 1 is also enriched by motifs corresponding to FOXO3, SOX7 and SRF and TEAD1, genes that are involved in regulation of apoptosis, in accordance with the functional enrichment of genes present in this cluster.

### Peaks of Cluster 3 frequently lie in promoter regions

**Fig. 7:**
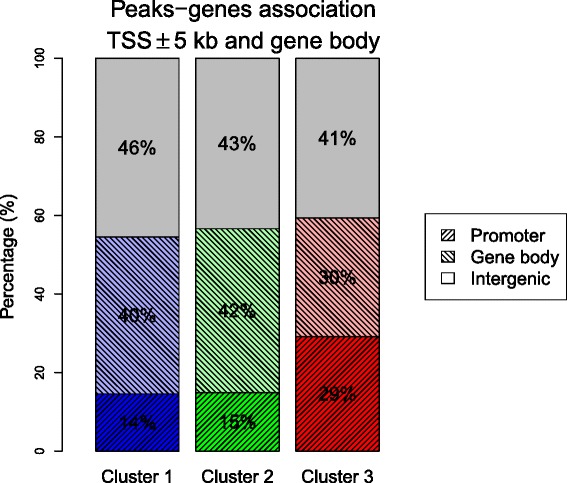
Association between GATA-1 peaks and genes for the three clusters in K562 cells. *Gray* areas show the intergenic peaks, peaks found in the promoter regions of a known gene (≤5 kb from the transcription start site) are in *dark colors*, and peaks located in a known gene body are in *light colors*. We observe that Cluster 3 is more associated to promoters than the other clusters (the *p*-values of the tests with alternative hypotheses that this proportion is greater than the one for Cluster 1 and 2 are 0). Results with less and more restrictive rules are shown in Additional file 1: Figure S8

Studying the association of GATA-1 peaks with known genes, we discover that about 55 % of peaks (7271 regions) are assigned to at least one gene and this percentage is similar in the three clusters (54 % in Cluster 1, 57 % in Cluster 2 and 59 % in Cluster 3). The clusters behave in the same way even considering the proportion of peaks associated to non-coding genes, as it is the same in all clusters (around 2 %). Interestingly, when we examine more deeply the genomic locations of the peaks, Cluster 3 stands out from the others because of its significantly higher association with promoters (~30 % of Cluster 3 compared to ~15 % of Cluster 1 and 2), defined as the regions within 5 kb upstream and downstream transcription start sites (Fig. 7). Testing the hypothesis that the proportion of peaks from Cluster 3 located in promoters is greater than the same proportion for peaks from Cluster 1 gives *p*-value = 0; we get the same *p*-value = 0 also considering Cluster 2, hence the association of Cluster 3 with promoter regions is statistically significant. Less and more restrictive definitions of promotor regions show the same association for Cluster 3 (Additional file 1: Figure S8).

### Cluster 2 is associated with a putative protein complex

To investigate the simultaneous binding of GATA-1 with other proteins, we consider a set of 237 publicly available ChIP-seq experiments for 95 different transcription factors, as well as 38 histone modification ChIP-seqs in K562 cells (from [17], see Additional file 2 for the detailed list of used datasets). Transcription factors ChIP-seq replicates are kept separated and MACS is used to call peaks, independently in each sample, using as control the same signal used with GATA-1 ChIP-seqs. In the case of histone modifications, we use peaks called by ENCODE. In addition, we also consider DNase I hypersensitive sites in K562 cells (GEO accession number GSM816655) in order to study open chromatin regions.

We use random forest classification, as explained in Methods, to select the experiments that are more correlated with our clusterization. Specifically, seven different analyses are performed, by using as response the clusters membership and alternatively classifying: 1) all clusters; 2) one cluster versus the union of the other two; 3) two clusters one against the other. All random forest models are able to predict a considerable portion of memberships, indicating that our peak shape clustering is related to co-located proteins. Notably, the overlaps of GATA-1 peaks with open chromatin regions stand out as important in all these analyses, according to Gini index (Additional file 2). Looking more deeply to the relationship between clusters and DNase-Seq regions, we can observe that the proportion of peaks that fall in accessible regions of the genome is typical of the different clusters. Indeed, almost all Cluster 2 peaks intersect DNase I hypersensitive sites (94 %), while the portion of peaks that fall in open chromatin regions is smaller in Cluster 3 (84 %) and it is further reduced in Cluster 1 (70 %). Anyway, the three proportions are much higher with respect to the random case (see Methods), in which only the 8 % of the peaks intersect open chromatin regions, supporting the claim that none of the clusters is composed exclusively by artifacts. Interestingly, the distribution of the percentage of intersection, conditionally to the intersection being non-zero, is essentially the same in all groups, and it is highly similar to the random case. Hence, the clusters are characterized by the proportion of peaks that overlap DNase I sites, rather than by the percentages of intersection (see Additional file 1: Table S5 and Figure S9). Inspecting the rankings of transcription factors and histone modifications ChIP-seqs, according to Gini index, we are able to identify a small set of regulatory elements that emerge as influent in the seven random forest classifiers we built, with all replicates in top positions (the complete rankings are reported in the Additional file 2). Specifically, we retain for further analyses all the proteins that are, simultaneously, 1) top 15 in at least three random forests; 2) top 30 in at least five random forests; 3) top 30 with all available replicates in at least 2 random forests. The eight transcription factors selected are GATA-2, CEBPD, HMGN3, TRIM28, PML, TAL-1, ZMIZ1 and CCNT2 (see Table 2 for the full protein names). Some of these proteins are known GATA-1 interactors. In particular, GATA-2 and TAL-1 can associate with GATA-1 in complex to regulate erythroid transcription. In addition, evidences of interaction between GATA-1 and PML have recently been shown [14]. We must point out that among these important regulatory elements there are no histone modifications. Indeed, if we perform similar random forest analyses using as predictors only the 237 ChIP-seqs for transcription factors, we get nearly the same results and the same accuracy in cluster membership predictions.

**Table 2:** Transcription factors related to the clustering

GATA-2	GATA binding protein 2
CEBPD	CCAAT/Enhancer-Binding Protein Delta
HMGN3	High Mobility Group Nucleosome-binding domain-containing protein 3
TRIM28	TRIpartite Motif-containing 28
PML	ProMyelocytic Leukemia protein
TAL-1	T-cell Acute Lymphocytic Leukemia protein 1
ZMIZ1	Zinc finger MIZ domain-containing protein 1
CCNT2	Cyclin-T2

The eight transcription factors emerged as relevant for the peak shape clustering in Replicate 1, according to Gini index in all the random forest classifiers built (experiments on K562 cells). Combinatorial interaction analysis reveals that Cluster 2 is characterized by the simultaneous binding of all these eight proteins, together with GATA-1

**Fig. 8:**
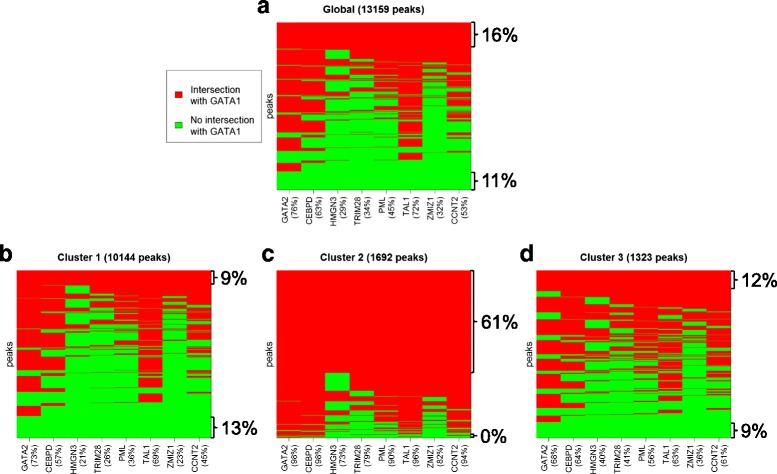
Combinatorial interaction analysis on the eight transcription factors selected with random forests (see Table 2), in Replicate 1 for K562 cells. Here we consider having an overlap when GATA-1 peak intersects at least one ChIP-seq replicate for the regulatory element of interest. **a** Results considering all the peaks simultaneously. **b**-**d** Results concerning the peaks that belong to three clusters separately. In all the plots, each row represents the overlap of a GATA-1 peak with the eight protein considered. The *red* color means that there is an intersection, while *green* stands for the absence of the protein in the correspondent GATA-1 region. The proportion of GATA-1 peaks that intersect the different proteins is indicated in brackets near the protein name. Results obtained considering the more stringent rule of having an overlap if the peak intersects  all replicates can be found in Additional file 1: Figure S10

The combinatorial interaction analysis on the transcription factors selected by using random forests shows that Cluster 2 is characterized by the simultaneous binding of all the eight transcription factors, in addition to GATA-1. The most significant results are obtained considering the intersections of GATA-1 peaks with at least one ChIP-seq replicate for the other regulatory elements under investigation. Nevertheless, the same conclusions are drawn even if we require that GATA-1 peaks overlap all available ChIP-seq replicates for the other proteins. Notably, the distribution of overlaps with a combination of these eight transcription factors in Cluster 1 and Cluster 3 is very similar to the global one, in which no combination outnumbers the others. On the contrary, ~61 % of the peaks belonging to Cluster 2 simultaneously intersect all the eight proteins considered, while only 4 regions contain GATA-1 alone (see Fig. 8 and Additional file 1: Figure S10).

The co-binding of these eight transcription factors in the genomic regions of Cluster 2 emerges also by using multiple correspondence analysis (see Methods). The advantage of this analysis is that it permits to study all the replicates simultaneously. In particular, by plotting the amount of total variation explained by an increasing number of principal coordinates (Additional file 1: Figures S11 and S12), we choose to focus on the first two components. The main effect of the first component (that explains, alone, ~40 % of the total variation in the data) is to contrast between the presence and the absence of overlaps, while the second dimension adds some variability among the different regulatory elements considered (Additional file 1: Figure S12). We observe that the various ChIP-seq replicates for the same protein are close in the space of the first two principal components, suggesting that they behave in a very similar way. Notably, Cluster 2 appears as highly different from the other two groups and the global case. Indeed, the first component conveniently separates Cluster 2 peaks from the others. Moreover, levels 1 (intersections with the considered ChIP-seq) of all variables are clustered together in Cluster 2, indicating high similarity between the peaks associated to this category.

### Genes associated to Cluster 2 tend to be underexpressed after knockdown of GATA-1

**Fig. 9:**
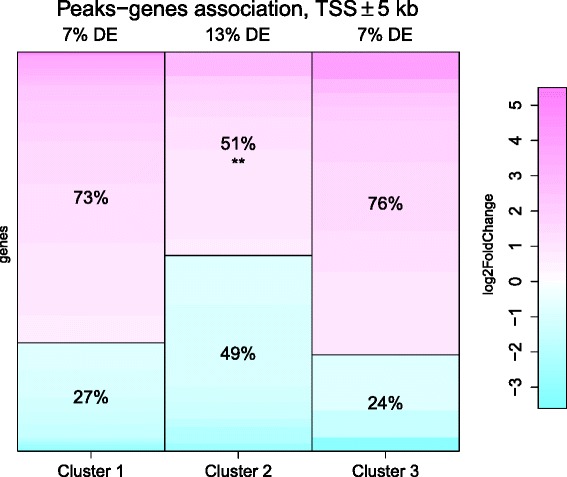
Gene expression analysis. Expression level fold change for differential expressed genes after knockdown of GATA-1 in K562 cells. The proportion of differential expressed genes in the different clusters is shown above each bar, where *purple* indicates overexpression, while *cyan* displays underexpressed genes. Only the known genes that are univocally associated to a single cluster are shown. Bonferroni corrected *p*-values of Fisher’s exact test on the percentage of overexpressed genes (as explained in Method) are shown with asterisks (with codes: <0.001 ‘***’, <0.01 ‘**’, <0.05 ‘*’). Gene expression analysis results obtained with less and more restrictive definition of promoter regions can be found in Additional file 1: Figure S15

To examine the potential correlation between cluster membership and gene expression, we associate each GATA-1 peak to a known gene if it falls within the region surrounding the transcription start site or in the gene body. Given that a peak can be assigned to more than one gene, and conversely each known gene can be associated to multiple peaks, some genes can turn out to be related to two or even all clusters (see Additional file 1: Figure S13a). In order to inspect possible relationships between peak shape and gene expression, we consider only the genes that are unambiguously associated to a single cluster in the next analyses. The gene expression experiments that we study in combination with the results of peak shape clustering consist in four publicly available RNA-seqs (RNA sequencings) in K562 cells [27] (GEO Accession number GSM798057 and GSM798058): two control samples and two replicates after independent knockdown for GATA-1. Details about the employed RNA-seq analysis pipeline can be found in Methods. Gene expression boxplots (Additional file 1: Figure S14a) show that there are small differences among the base expression level (RNA-seq without any treatment) of the genes associated to different clusters. Indeed, RPKM in Cluster 3 is slightly higher and less variable then the other clusters, while Cluster 1 shows lower and more variable RPKM. When we focus on the genes that are differentially expressed after knockdown of GATA-1, Cluster 2 exhibits considerable peculiarities (Fig. 9). Specifically, Cluster 2 genes are more differentially expressed than genes associated to the other two clusters (13 % versus 7 % for Cluster 1 and Cluster 3). About 27 % of the differentially expressed genes that are associated to at least one GATA-1 peak are down-regulated following GATA-1 knockdown. This percentage remains almost the same if we analyze only the genes univocally associated to Cluster 1 or Cluster 3 (27 % and 24 %, respectively). Conversely, half of the genes assigned to Cluster 2 (49 %) are down-regulated. Therefore the fraction of genes associated to Cluster 2 that are down-regulated after silencing of GATA-1 is almost double than the ones for the other two clusters and for the whole set of differentially expressed genes bound by GATA-1 (Bonferroni corrected *p*-value of Fisher’s exact test on the percentage of overexpressed genes in Cluster 2 is equal to 0.0015). This result suggests that GATA-1 in peaks of Cluster 2 acts more prevalently as a transcriptional activator. The reported results are robust to the peak-gene assignment rule: we obtain the same conclusions even considering different definitions of promoter regions (≤10 kb or ≤2.5 from the TSS). The detailed results can be found in Additional file 1: Figure S13-S15.

### GATA-1 in Megakaryocytes

In order to check whether our findings hold in another biological system, we decide to redo the analysis for the transcription factor GATA-1 in primary human megakaryocyte cultures. The advantage to study GATA-1 binding in primary human megakaryocytes is that they represent an additional and more relevant biological model of the *in vivo* situation. In addition, we analyze an analogous ChIP-seq in primary mouse megakaryocytes, to understand whether GATA-1 peak shapes are maintained across different organisms.

### Three clusters for GATA-1 peaks in human megakaryocytes

We consider a publicly available ChIP-seq experiment by Tijssen et al. [24] (GEO accession number GSE24674), on which we apply part of the analysis pipeline described in Methods (data are not enough for robustness analysis, and validation is done directly by using the experiments and results reported in the paper by Tijssen and coauthors). The sequenced reads are preprocessed as detailed in Methods. Briefly, BWA is used to map high quality reads to the human reference genome hg19. Afterward, MACS is employed to call peaks, obtaining 3399 GATA-1 peaks (after blacklisted regions filtering). The estimated fragments length of 57 nucleotides is then used to compute the coverage function for each peak.

**Fig. 10:**
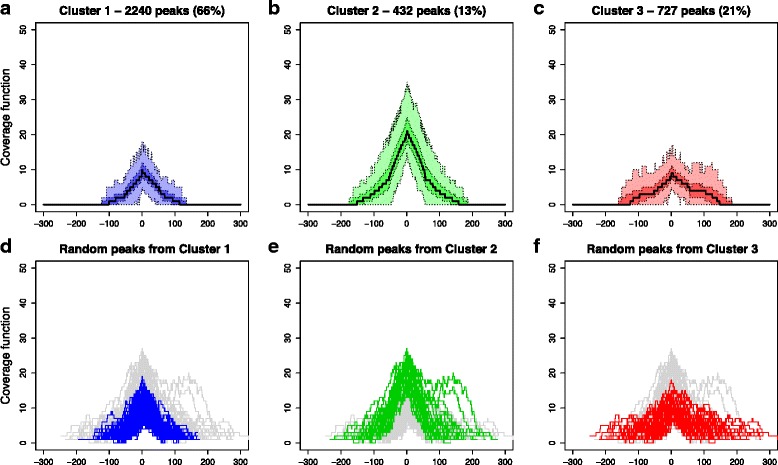
The three clusters obtained on shape indices in megakaryocytes. Results of *k*-mean algorithm with Euclidean distance on the standardized shape indices, in megakaryocytes. **a**-**c** Pointwise boxplots of the coverage function in the three clusters. For each abscissa, *black* indicates the median value, *dark colors* highlight the central 50 % of the distribution, while *light colors* correspond to the boxplot whiskers. **d**-**f** A random sample of 200 peaks (for visualization reason), with colors highlighting the cluster membership. In both images, peaks are registered using as landmark the location of their maximum height

Similarly to what we obtained with GATA-1 in K562, also in megakaryocytes the multivariate analysis on shape indices leads to three clusters (see the total within-clusters sum of squares plot in Additional file 1: Figure 16a). Notably, examining the nature of the resulting clusters, we conclude that the selected shapes have the same characteristics of the ones selected within K562 peaks, with the only difference being the proportions of peaks in the different clusters. Clustering results are shown in Additional file 1: Figures S16b-S17 and Fig. 10 (that report the scatterplot of indices, their distributions and the coverage function in the different groups), where for the sake of clarity the clusters are named and colored as their matching pair in K562. In summary, Cluster 1 includes the majority of the peaks (~66 %), that are bell-shaped and quite low. Cluster 2 is the smallest group (~13 % of the peaks) and is composed by high, bell-shaped peaks. Finally, Cluster 3 comprises the most complex and wide peaks (~21 % of GATA-1 regions).

### Key hematopoietic transcription factors simultaneously bind regions of Cluster 2

**Fig. 11:**
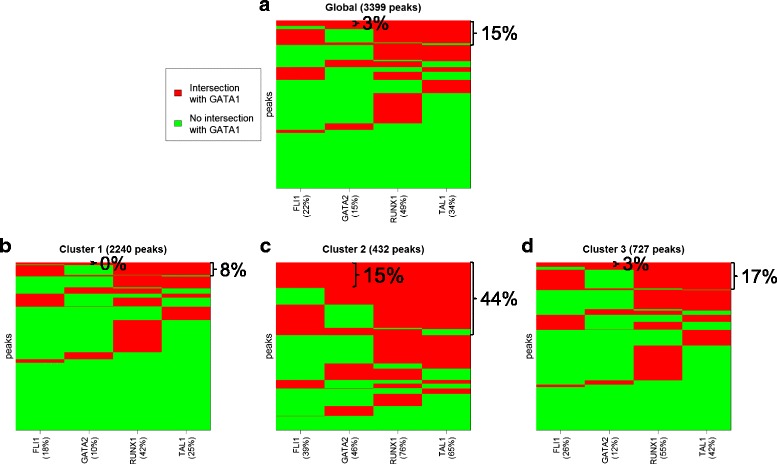
Combinatorial interaction analysis on the five hematopoietic transcription factors studied in human megakaryocytes. **a** Results considering all the peaks simultaneously. **b**-**d** Results concerning the peaks that belong to three clusters separately. In all the plots, each row represents the overlap of a GATA-1 peak with the eight protein considered. The *red* color means that there is an intersection, while *green* stands for the absence of the protein in the correspondent GATA-1 region

To study the genome-wide binding of multiple transcription factors and identify regulatory complexes, Tijssen et al. generated ChIP-seqs for the hematopoietic transcription factors GATA-1, FLI1, GATA-2, RUNX1 and TAL-1. By analyzing all the regions bound by at least one of these proteins, the authors showed that the five transcription factors in megakaryocytes bind the DNA together more frequently than expected at random [24]. To examine the potential relationship between the three peak shape clusters and the binding of GATA-1 with different sets of proteins, we pre-process the four ChIP-seq experiments available (GEO accession number GSE24674) and we use MACS to identify the binding sites of each transcription factor (see Methods). Afterwards, we perform combinatorial interaction analysis on the whole set of GATA-1 peaks, as well as on the three clusters separately. We observe (see Fig. 11) that only Cluster 2 is characterized by the simultaneous presence of all the five proteins. Notably, about 15 % of the peaks belonging to Cluster 2 overlap all five transcription factors together, while this co-binding is present in only ~1 % and ~3 % of peaks from Cluster 1 and Cluster 3, respectively. Moreover, at least three proteins among FLI1, GATA-2, RUNX1 and TAL-1 simultaneously bind only ~15 % of GATA-1 sites, but this combination is present in ~44 % of peaks in Cluster 2. These findings suggest that we can actually use GATA-1 peak shape to highlight the co-localization preference of the five hematopoietic transcription factors in megakaryocytes. Furthermore, 5 of the 6 genes whose depletion resulted in a severe hematological phenotype in zebrafish (i.e. MARCH2, MAX SMOX, EMILIN1 and SUFU), that were identified by the co-localization of the five TFs in [24], are part of Cluster 2. Thus, peak shape conveys biologically relevant findings.

### GATA-1 peaks in mouse and human megakaryocytes have similar shapes

We apply our analysis pipeline to an ENCODE ChIP-seq for GATA-1 in mouse megakaryocytes (GEO Accession number GSM923586 Replicate 1, antibody used: sc-265, Santa Cruz Biotech) [16]. We start our preprocessing on the reads already aligned to mm9 reference genome, then we call peaks using MACS as detailed in Methods. After filtering our blacklisted regions we obtain 2586 peaks and an estimated fragments length of 52 nucleotides, comparable with the length obtained in human megakaryocytes. The clustering of shape indices produces four clusters in this case. However, one cluster is made of only 13 peaks, and 10 out of 13 lie in repetitive regions (they have more than 80 % overlap with RepeatMasker track), thus they are false positive peaks. Importantly, these 13 peaks show extreme shapes (they are quite high, wide, multimodal and really complex peaks) and our clustering methodology is able to recognize and group them together. After excluding this artifact cluster from further analyses, GATA-1 in mouse and human megakaryocytes have the same number of clusters, with very similar shapes and indices distributions. The sole difference we can spot is that mouse Cluster 2 peaks are a bit more complex than human Cluster 2 peaks. This result suggests that peak shape might be the highly similar in the same cell types of different organisms.

### Clustering of shape indices for other ChIP-seq experiments

We examine a set of ChIP-seqs for nine different proteins in K562 cells (see Additional file 4 for details), to understand whether the clustering of shape indices always give rise to the same number of clusters and to the same shapes that are found in GATA-1. All the analyses are performed using the same methodology described in Methods. Interestingly, we observe that the number of clusters varies depending on the protein under consideration. In particular, we obtain four clusters for TRIM28, CCNT2, Z-MIZ1 and PML, and three clusters for GATA-2, TAL-1, c-Fos, SP1 and NF-YA. Differences in the number of clusters depend on the complexity of the data under analysis. Indeed, in all the experiments that give rise to four clusters, one of them is quite small (less than 6 % of peaks belong to it) and contains broad and complex peaks; we exclude that these peaks are alignment artifacts (as we observe for GATA-1 in mouse megakaryocytes) because they are not located in repetitive regions and they show a central stronger signal. In these cases the peak shapes appear to be generally more complex than the shapes we obtained in GATA-1 (Additional file 1: Figures S18-S20 show the representative example of CCNT2 peak shape clustering). Moreover, different TFs have various rates of peaks in the different clusters. Among the TFs with three clusters, we observe that some proteins (such as GATA-2, c-Fos and SP1) produce almost the same clustering of GATA-1, while other TFs (TAL-1 and NF-YA) have slightly more complex shapes, with characteristic rates (see Additional file 4).

## Conclusions

We have developed a novel analysis method that studies ChIP-seq enriched regions focusing both on the complexity and the signal intensity of peaks. Shape Index Clustering for ChIP-seq peaks has the ability to identify different groups of peaks in a single ChIP-seq, based on differences in peak shape. These differences could not be identified using either tag counts or peak enrichment alone. In addition, the proposed pipeline involves several downstream analyses able to investigate possible relationships between the peak shape clusters identified by SIC-ChIP and biological properties.

By applying the proposed analysis pipeline to ChIP-seq experiments for the transcription factor GATA-1 in K562 cells and in primary human megakaryocytes, we have demonstrated that statistically significant different peak shapes are correlated with several cooperative transcriptional regulators. We have shown that GATA-1 peak shape is associated with characteristic regulatory complexes and changes in gene expression profiles. Moreover, peak shape can shed light on previously described GATA-1 occupancy profiles. Specifically, considering GATA-1 ChIP-seqs in K562 cells, peaks belonging to Cluster 2 emerge as part of a putative protein complex that comprises well known GATA-1 interactors such as GATA-2 and TAL-1. The target genes of these peaks appear to be mostly down-regulated after knockdown of GATA-1, suggesting that GATA-1 in Cluster 2 behaves primarily as a transcriptional activator. This result is in agreement with previous studies that reported the ability of GATA-1 to act both as an activator and as a repressor, and that also highlighted a positive correlation between activated GATA-1 target genes and binding of TAL-1 [28, 29]. In conclusion, our study demonstrates that ChIP-seq shapes include information regarding the binding of other proteins beside the one used for precipitation and it is correlated with gene expression. Moreover, studying other ChIP-seq experiments with the same methodology, we showed that peak shape clustering depends on the protein under investigation. Thus, ChIP-seq profiles carry much more information than previously suspected.

Although we presented our methodology applied mainly to ChIP-seq experiments for transcription factors, the same principles can be applied to the investigation of other ChIP-seq data, e.g. histone modification peaks. Furthermore, a generalization of these methods may be employed to analyze ChIP-exo data [30]. We expect that applying the “peak shape concept” to ChIP-exo peaks can lead to even more interesting and clear correlations between shape and biological properties, thanks to the high resolution reached by this technique.

## Methods

### ChIP-seq pre-processing

#### Reads alignment and filtering

Mapping reads back on a reference genome is the first pre-processing step that must be done when analyzing ChIP-seq data. We perform it by using Burrows-Wheeler Aligner (BWA) [31], unless we are dealing with ENCODE ChIP-seqs. In this case, ENCODE mapping is taken as the starting point of the analysis pipeline. In all cases the experiments are made up of single-end reads and only high quality tags that maps uniquely to the genome are retained for further analysis. Moreover, only autosomes and X chromosome are considered. Reads duplicates are discarded too.

#### Peak calling

The peak caller MACS [18], which is one of the best ChIP-seq callers [32] and optimally estimates the spatial resolutions of binding events [2], is run with the aim of detecting significantly enriched regions in the genome (namely the peaks) with respect to a control signal. Peaks represent the areas where the protein of interest interacts with the DNA. We use MACS default options except for the p-value cutoff for peak detection, that we set to 1e-8 (more stringent than the default 1e-5). In this step the average length of the initial fragments from which tags are sequenced is estimated from reads positions in the two DNA strands.

#### Filtering of blacklisted regions

The ENCODE Data Analysis Consortium Blacklisted regions [16] (a set of artifact regions in the genome), is used to filter the resultant peaks, in order to obtain a purified collection of peaks.

#### Coverage function computation

Short reads are extended in 3' direction to the average fragment length estimated before. The coverage function, defined as the base by base count of the elongated reads, is then computed.

### Multivariate analysis on shape indices

#### Index computation

Consider the genomic region *R = {x*_*1*_*,…,x*_*L*_*}* of *L* contiguous nucleotide positions found by the peak caller. We define the corresponding peak as the function *f* that associates to each nucleotide *x*_*i*_ the coverage function, i.e. the count of elongated tags, calculated at that position *f(x*_*i*_*)*. We summarize the shape of each peak with five indices: the first two are related to the intensity of the signal, while the others are connected with the complexity of the peak (Fig. 3). In particular, for each peak we calculate:

1. The *maximum height* of the peak, i.e. *h =* max_*xi ∈ R*_*f(x*_*i*_*)*;
2. The *area* subtended by the function, i.e. *A = *∑_*xi ∈ R*_*f(x*_*i*_*)*;
3. The *full width at half maximum*, that is the width of the peak (the projection on genome positions) at half of its maximum height, i.e. *w*_*h/2*_ *=* max *G -* min *G*, where *G = {x*_*i*_*∈ R : f(x*_*i*_*) ≥ h/2}*;
4. The *number of local peaks p*_*local*_ of the smoothed function, as detailed below;
5. The *shape index M* (computed as explained below), divided by the maximum height of the peak.

To calculate the number of local peaks *p*_*local*_ we need, first of all, to smooth the function *f* in order to filter out noise (Fig. 3a). A cubic B-splines basis with knots every 20 nucleotides is fitted by using ordinary least squares (see, e.g., [33]). The index is the number of local maxima of the resulting smoothed function, provided that they are at least 50 nucleotides apart and their difference in height from the two nearest local minima is more than the 20 % of the maximum height of the peak.

The shape index *M* is a measure of the complexity of the peak that is robust to noise, computed as suggested in [6, 34]. Each peak is associated with a rooted tree, built by following the profile of its function *f*. In particular, we start with constructing the root of the tree. Then we look at the value of *f* at the first nucleotide *x*_*1*_ and we create a new node of depth *f(x*_*1*_*)*. At this point, for *i ∈ 2,…,L*, the nucleotide *x*_*i*_ is considered. A new node is created in correspondence to an increase of the function (when *f(x*_*i*_*) > f(x*_*i-1*_*)*). When the function decreases (that is *f(x*_*i*_*) < f(x*_*i-1*_*)*), we move toward the root to the parent of the current node. Finally, if the function keeps constant (we have *f(x*_*i*_*) = f(x*_*i-1*_*)*), nothing is done. An example of a peak and the corresponding tree resulting from this procedure is shown in Fig. 3b. The index *M* is the number of edges in a maximal matching for the constructed tree, that is the highest number of edges of the tree without common nodes. It is clearly extremely dependent on the height of the tree, that turns out to be the maximum height of the peak. Consequently, we consider the index *M* divided by the maximum height *h*. The resulting index is related to the complexity of the peak meaning that, height being equal, it is bigger when the peak is multimodal so that the tree has multiple branches. In addition, note that noise in the peak converts to high degree nodes, hence it does not affect the maximal matching for the tree.

#### Clustering

We use the *k*-mean algorithm with Euclidean distance on the five standardized indices to cluster the peaks (see, e.g., [35, 36] for details on *k*-means as well as other multivariate clustering techniques). In this step, each replicate is considered separately, with the aim of finding statistically significant differences in peak shape inside a single ChIP-seq. The “correct” number of clusters *k* is estimated through the analysis of total within-clusters sum of squares plot. Each resulting cluster is characterized by a specific distribution of the five indices, representing its typical shape. This characterization is illustrated by the scatterplot of the indices and by the scatterplots on the first components obtained with PCA and ICA, all colored according to the clustering obtained with *k*-means. Moreover, the different peak shapes in the resulting clusters are shown by boxplots of the shape indices and pointwise boxplots of the coverage function in the different clusters, besides the plots of a random sample of peaks.

#### Robustness analysis

We take advantage of the multiple replicates, when they are available, to evaluate the robustness of the proposed technique. First, only peaks that are present in all replicates are selected. The overlapping regions are defined as the contigs of the peaks in all replicates, that is the union of the genomic areas corresponding to peaks with non-empty intersection in different replicates. Each of these regions *R = {x*_*1*_*,…,x*_*L*_*}* is associated with its corresponding peaks, i.e. the coverage functions *f*_*j*_ for each replicate *j = 1,…,J*. Intensity and shape indices are computed on all peaks as explained above. Subsequently, the peaks are clustered independently in each replicate, as illustrated in the last paragraph. Finally, the robustness of the method is evaluated by means of the correspondences between cluster memberships of a peak in the different replicates.

### Validation and characterization of clusters

#### Gene ontology analysis

Gene Ontology and other annotation ontologies enrichment analysis is done by using GREAT version 2.0.2 [37] with default association rule, for the whole set of ChIP-seq peaks found, as well as for the peaks in each cluster. This study permits to correlate clusters, and consequently peak shape, with an inferred biological meaning.

#### Motif analysis

Next step consists in different types of motif analysis. De novo motif discovery and motif enrichment analysis are performed with MEME-ChIP version 4.9.1 [38] (with default parameters), to provide a comprehensive view of the sequence motifs under the peaks in each cluster. Furthermore, we also run this tool after sampling the same number of peaks from each cluster, in order to compare the significance of the motifs found in the different groups. Afterwards, we analyze motif occurrences in order to understand how many times the same motif is present under each peak. We also compute, in each cluster, the distribution of motif distance from peak maximum (when more than one motif is found under a single peak, we consider the lowest distance of the motifs from the maximum). This points out whether the motif is central in the peak or it is a side motif.

#### Peaks localization

To investigate the genomic locations of the different clusters, we annotate each ChIP-seq peak as lying in a promoter, in a gene body or in a intergenic region. We define the promoter region as the area ≤ 2.5 kb, ≤ 5 kb or ≤ 10 kb from a transcription start site, using RefGene annotation database and considering both coding and non-coding genes. Plots and hypothesis tests are used to evaluate whether any difference in the proportion of peaks associated to genes in the different clusters exists, either in promoters or in gene bodies. Moreover, the potential association of the clusters with a specific gene type (coding or non-coding) is inspected too.

#### Analysis of overlap with transcription factors, open chromatin regions and histone modifications

An important characterization of a group of ChIP-seq peaks is given by their intersection with other transcription factors, indicating the co-occurrence of different protein bindings in the same site and suggesting the existence of protein complexes or co-regulatory activities. Relationships with chromatin accessibility and histone modifications are also significant. In particular, we observe that transcription factors are more likely to bind DNA in open chromatin regions. Therefore, DNase I hypersensitive sites are considered and used to compute the proportion of peaks in each cluster that overlap open chromatin regions. The distribution of the percentage of intersection, conditionally to be non-zero, is evaluated too. Both are compared to the random case, obtained by randomly shuffling the peaks among the chromosome where they lie, after excluding repetitive elements given by the RepeatMasker track of UCSC [39]. This allows us to characterize the different clusters, excluding at the same time that the procedure described in the previous subsection selects some clusters of noisy regions with artifact signals.

Moreover, we use random forest analysis (see [40] for details about this method) to assess which co-occurrences are more correlated with the regions of interest and to select the most important regulatory elements in relation with the clusters identified as explained before. Specifically, for each single peak of the starting ChIP-seq, we compute the percentage of intersection with the set of regions selected by a ChIP-seq for a different transcription factor or histone modification. Doing this calculation for all the available proteins, we obtain a matrix *P* whose rows represent the different peaks, while the columns correspond to the other considered experiments. We add also a column with the percentage of intersection with DNase I hypersensitive sites. The various ChIP-seq (and DNase-Seq) overlaps are the predictors of a random forest classification with response the categorical variable of clusters membership. Priors for the classes are used to handle imbalanced classification problems. Once the random forest classifier is built, variable importance is estimated through the mean decrease in Gini index [40, 41]. Briefly, for each node within a tree of the random forest classifier, the Gini index is computed as *1-p*^*2*^*(c*_*1*_*)-…-p*^*2*^*(c*_*k*_*)* where *p*^*2*^*(c*_*i*_*)* is the proportion of the samples assigned to the node belonging to category *c*_*i*_. Every time a node is splitted using a certain variable, there is a decrease in Gini index. The variable total decrease is given by the sum of these Gini index reductions over all nodes of a tree in which the variable is used to split. Finally, the mean decrease in Gini index is obtained averaging over all the trees. By ranking regulatory elements according to their importance in classifying all the different clusters, or one cluster against the others, and by looking at concordance between replicates, we can select a small number *N* of proteins on which to implement what we term *combinatorial interaction analysis*.

The combinatorial interaction analysis consists of evaluating the number (and the percentage) of peaks of the starting ChIP-seq that overlap each of the 2^*N*^ possible combinations of the transcription factors and histone modifications selected by using random forests. The same count is done for the different clusters obtained in the previous steps. Here we can say that a peak of the starting ChIP-seq intersects a regulatory element when it overlaps at least one ChIP-seq replicate for that protein (less stringent request) or when it overlaps all replicates (more stringent rule). In both cases we require a minimum overlap of 1 bp. Here we are interested in differences between clusters regarding specific co-occurrences, that can give a functional explanation to the statistically significant different shapes found.

Lastly, after converting the matrix *P* of the percentage of intersections to a boolean matrix that indicates overlap versus non-overlap, multiple correspondence analysis (see, for example, [42] for a detailed explanation of this technique) is used to study the relationships between overlaps with different proteins, by evaluating all the replicates simultaneously. The map showing the different levels of each variable in principal coordinates allows us to assess the similarity between ChIP-seq replicates for the same protein and to detect associations in overlapping elements in the different clusters obtained by using the methodology we proposed before.

#### Gene differential expression analysis

To assess whether or not peak shape is related to the regulation of gene expression, we combine the results of previous clustering and RNA-seq experiments. We assign each ChIP-seq peak to a known gene (using RefGene database) if the peak is located less than 5 kb from the transcription start site of the gene or if it falls within the gene body. We inspect less and more restrictive association rules too, considering peaks located less than 10 kb or 2.5 kb from the transcription start site, besides gene body. Once the association is established, we inspect the possible correlations between clusters and gene expressions by plotting in logarithmic scale the reads per kilobase per million (RPMK), computed normalizing HTSeq count results on a RNA-Seq without treatment. Moreover, we are interested in recognizing peak shapes involved in regulating some genes targeted by the protein of interest. This goal is achieved by analyzing changes of gene expression in a RNA-Seq after knockdown of the protein under investigation. After running DESeq with default options to select significantly differential expressed genes (the ones with false discovery rate < 0.05), we analyze the percentages of up and down regulated genes in the silenced cells, for each cluster. This permits to identify shapes that are more related to the protein acting as repressor or activator, respectively. To assess the significance of the findings, Fisher’s exact test is performed to test the null hypothesis of independence between this percentages and the association of the genes with a particular cluster, versus the alternative hypothesis that the odd ratio is less than one. Resulting p-values are then corrected for multiple testing by using Bonferroni method. To avoid mixing of different clusters, in these analyses we contemplate only the genes that are univocally associated to a single cluster.

## Additional files

Additional file 1:
**Supplementary Figures and Tables.** (PDF 2223 kb) Additional file 2:
**Random forest analysis.** (XLSX 74 kb) Additional file 3:
**GREAT GO Biological Process.** (XLSX 364 kb) Additional file 4:
**ChIP-seq data.** (XLSX 14 kb)

## Abbreviations

ChIP-exo: Chromatin ImmunoPrecipitation exonuclease
ChIP-seq: Chromatin ImmunoPrecipitation sequencing
DNase-seq: DNase I hypersensitive sites sequencing
GO: Gene Ontology
RNA-seq: RNA sequencing
RPKM: Reads Per Kilobase per Million
TF: Transcription Factor
