# Supplementary material for: Peak shape clustering reveals biological insights
Source: BMC Bioinformatics. 2015 Oct 28;16:349. doi: 10.1186/s12859-015-0787-6 (PMC4625869; doi:10.1186/s12859-015-0787-6)
Supplement: Additional file 1: — Supplementary Figures and Tables. (PDF 2223 kb) [file 12859_2015_787_MOESM1_ESM.pdf]

Peak shape clustering reveals biological insights – Additional file 1

Marzia A Cremona[1], Laura M Sangalli[1], Simone Vantini[1], Gaetano I Dellino[2,3], Pier Giuseppe Pelicci[2,3], Piercesare Secchi[1], and Laura Riva[4,*]

[1] MOX - Dipartimento di Matematica, Politecnico di Milano, Milano, Italy

[2] Department of Experimental Oncology, European Institute of Oncology, Milano, Italy

[3] Dipartimento di Scienze della salute, Università degli Studi di Milano, Milano, Italy

[4] Center for Genomic Science of IIT@SEMM, Fondazione Istituto Italiano di Tecnologia, Milano, Italy

* Corresponding author: Laura Riva laura.riva@iit.it

Emails:

Marzia A Cremona marziaangela.cremona@polimi.it

Laura M Sangalli laura.sangalli@polimi.it

Simone Vantini simone.vantini@polimi.it

Gaetano I Dellino gaetano.dellino@ieo.eu

Pier Giuseppe Pelicci piergiuseppe.pelicci@ieo.eu

Piercesare Secchi piercesare.secchi@polimi.it

Laura Riva laura.riva@iit.it

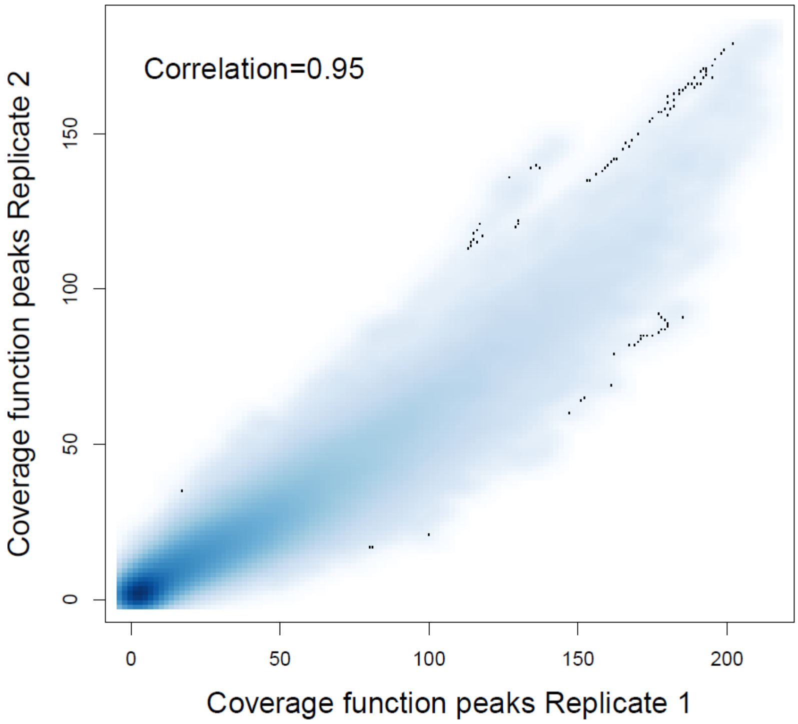

Figure S1. Correlation between replicates. Smoothed scatterplot of the coverage functions in Replicate 1 and Replicate 2 for GATA-1 in K562 cells. Here we evaluate the coverage functions on all nucleotides that fall in common peaks.

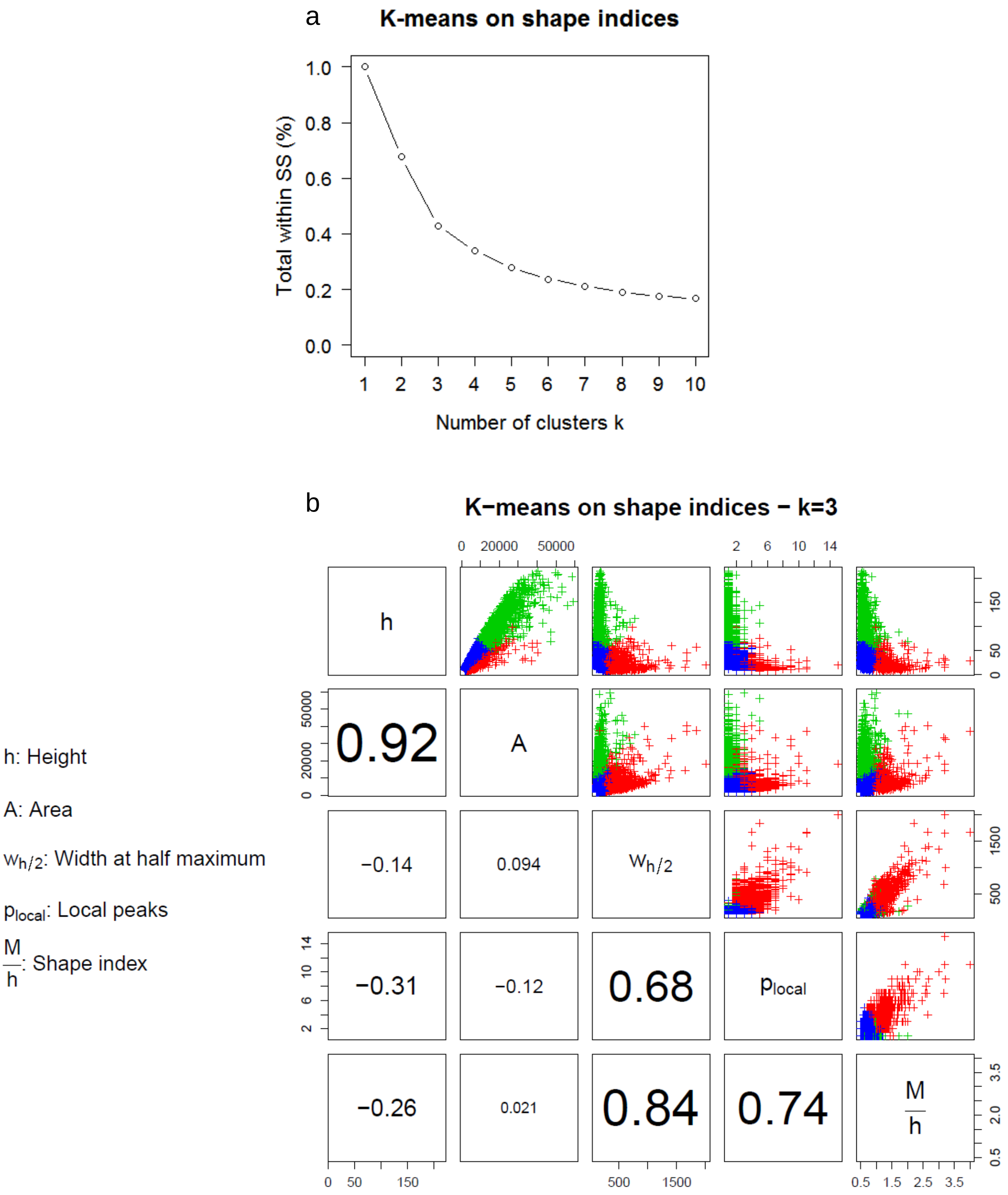

Figure S2. K-means results. Results of *k*-mean algorithm with Euclidean distance on the standardized shape indices, in Replicate 1 for K562 cells. (a) The total within-clusters sum of squares plot suggests that *k*=3 is the correct number of clusters. (b) Scatterplot of the five indices, with elements colored according the cluster they belong to after choosing *k*=3 in the *k*-mean results. The lower panels show the correlations between the different indices, considering all the peaks at the same time.

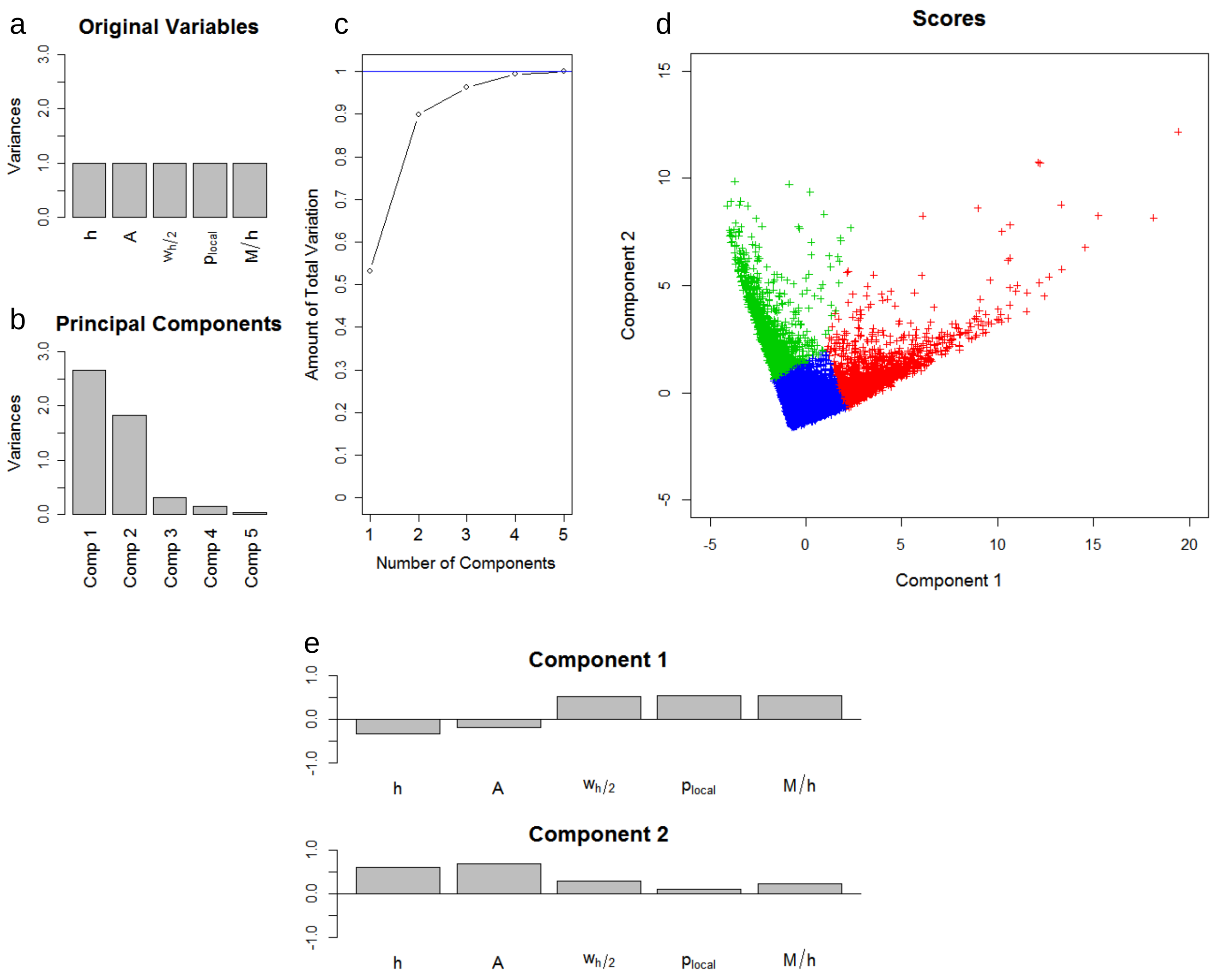

Figure S3. Principal Component Analysis. Results of Principal Component Analysis on the five standardized shape indices, in Replicate 1 for K562 cells. (a) The variances of the initial standardized indices. (b) The variances of the principal components obtained by Principal Component Analysis. (c) Plot of the amount of total variation explained by the first n principal components; we observe that the first two components together explain about the 90% of the variability in the data. (d) Scatterplot of the data in the plane defined by the first two principal components, with each point representing a peak colored according to the results of *k*-mean clustering with *k*=3, as presented in Additional file 1: Figure S2. (e) The first two components in terms of the initial shape indices.

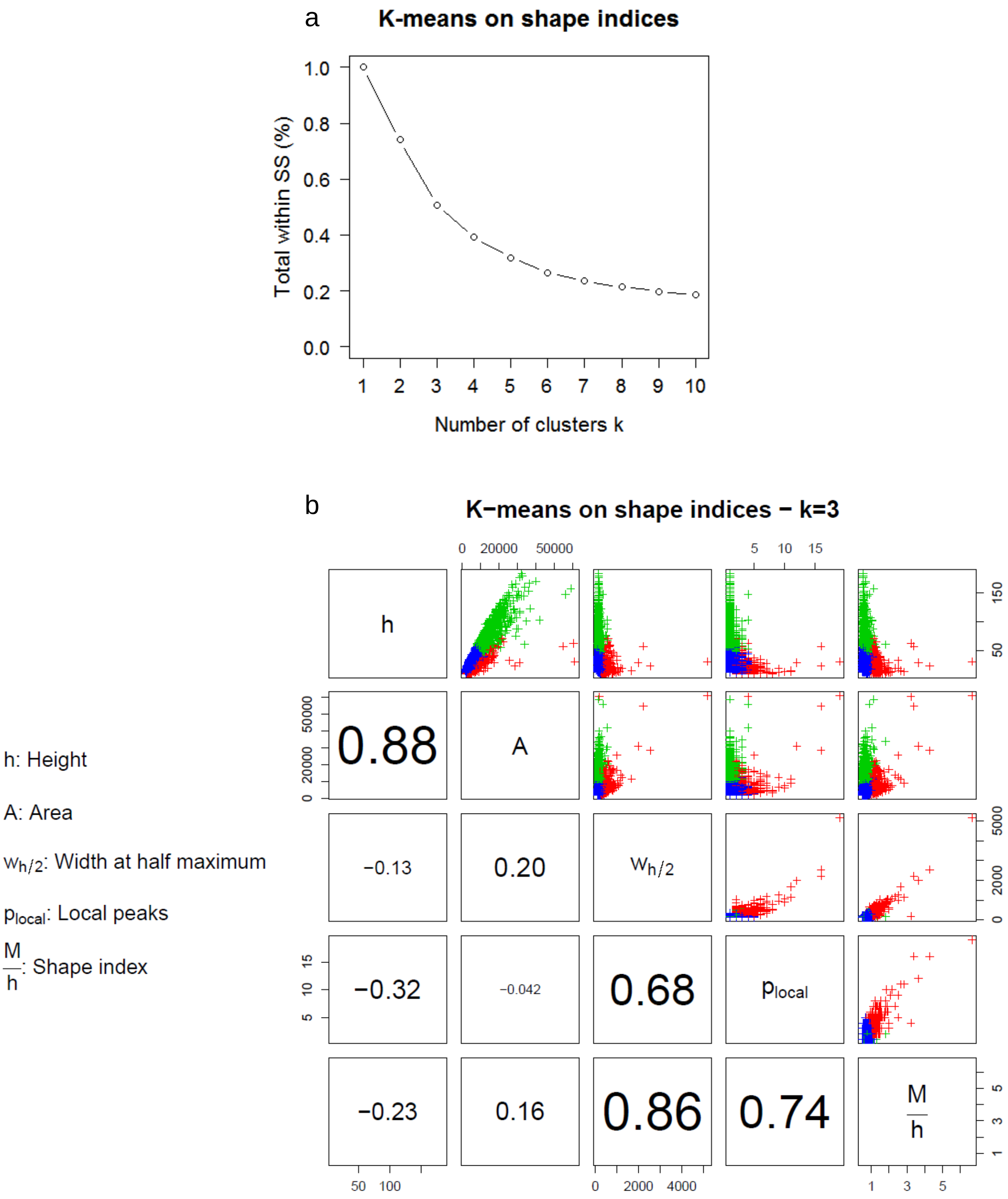

Figure S4. K-means results for Replicate 2. Results of *k*-mean algorithm with Euclidean distance on the five standardized shape indices, in Replicate 2 for K562 cells. (a) The total within-clusters sum of squares plot suggests that *k*=3 is the correct number of clusters. (b) Scatterplot of the five indices, with elements colored according the cluster they belong to after choosing *k*=3 in the *k*-mean results. The lower panels show the correlations between the different indices, considering all

the peaks at the same time. *K*-mean results on Replicate 2 look very similar to the ones obtained with Replicate 1, shown in Additional file 1: Figure S2.

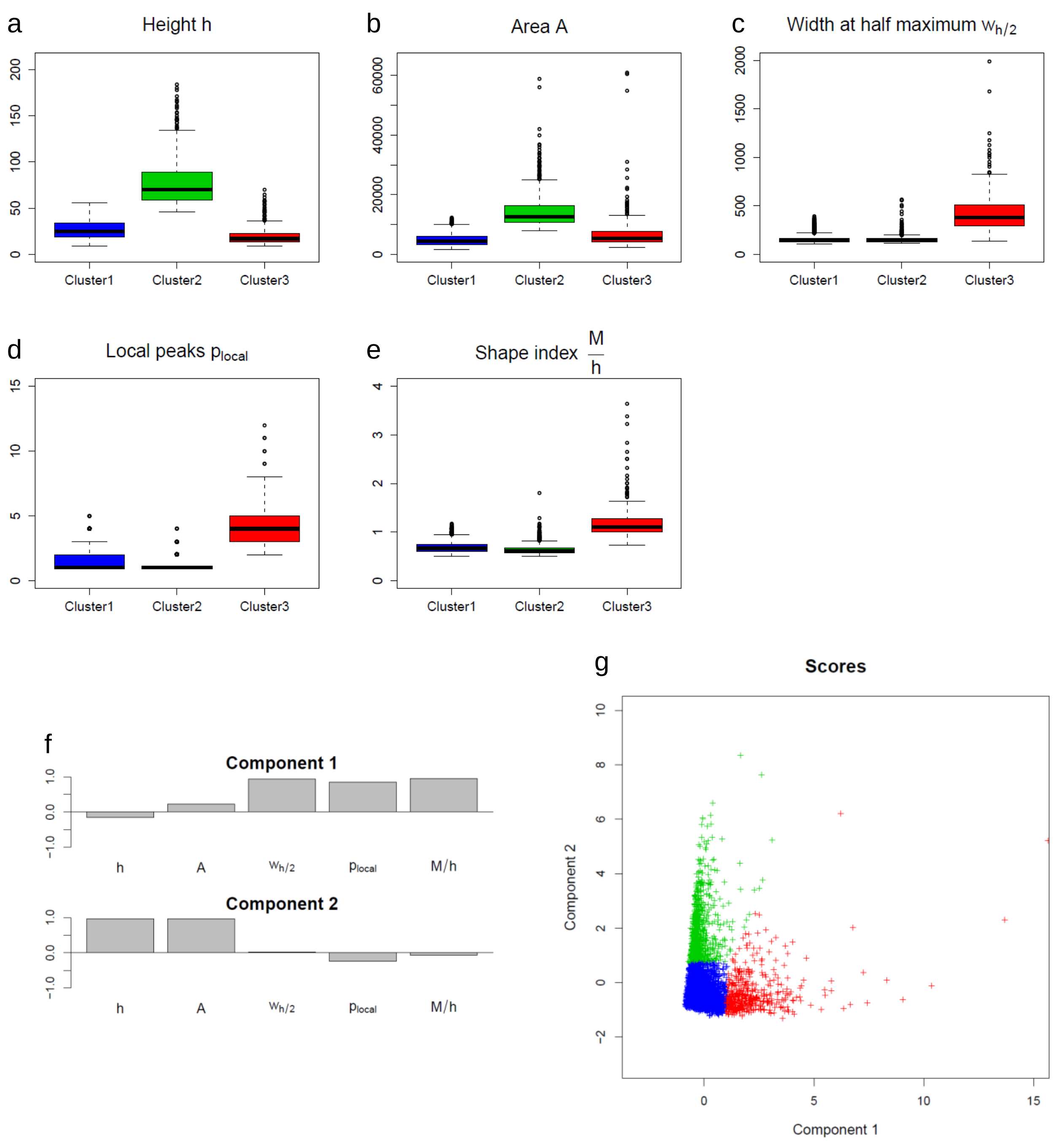

Figure S5. Distribution of shape indices in the three clusters for Replicate 2. Results of *k*-mean algorithm with Euclidean distance on the standardized shape indices, in Replicate 2 for K562 cells. (a-e) Shape indices boxplots in the three clusters. (f) The first two components obtained with independent component analysis, in term of the initial shape indices. (g) Scatterplot of the data in the plane defined by the first two independent components, with each point representing a peak and colored according to the cluster it belongs to. These plots look very similar to the ones obtained with Replicate 1, shown in Fig. 4.

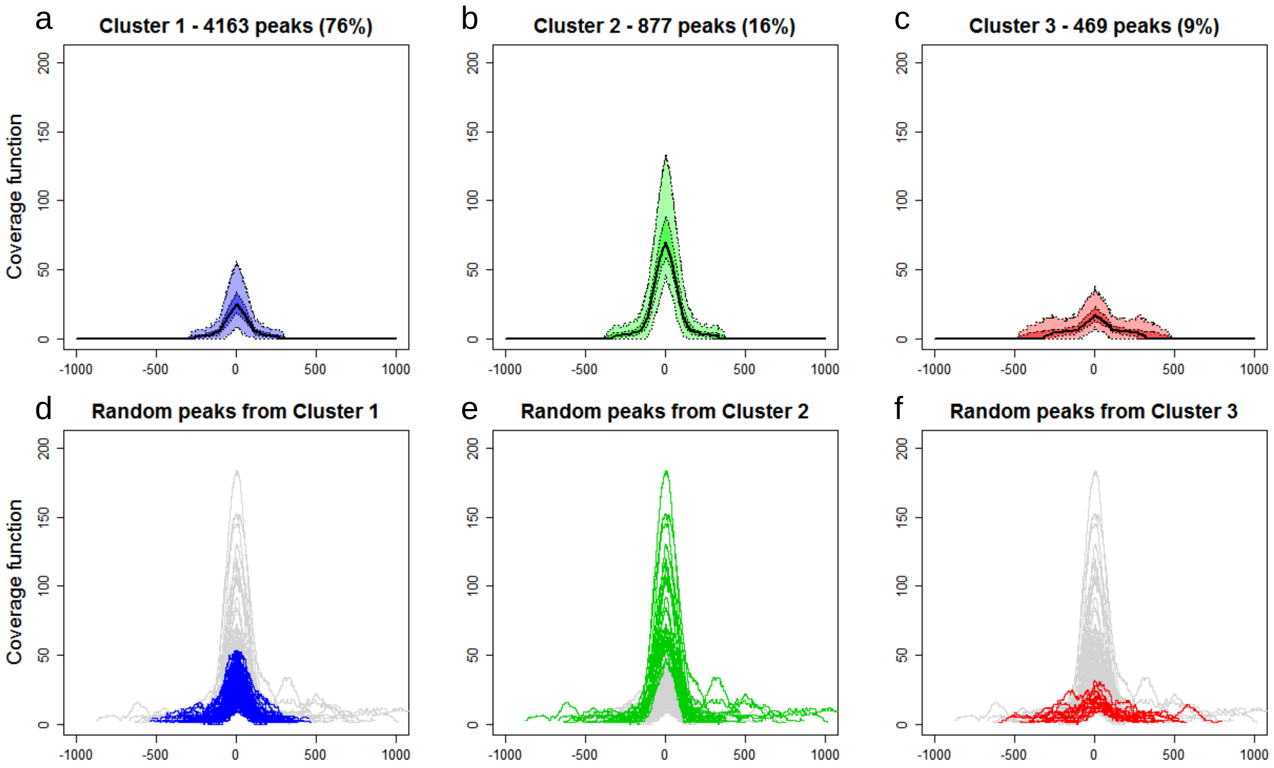

Figure S6. The three clusters obtained on shape indices for Replicate 2. Results of k-mean algorithm with Euclidean distance on the standardized shape indices, in Replicate 2 for K562 cells. (a-c) Pointwise boxplots of the coverage function in the three clusters. For each abscissa, black indicates the median value, dark colors highlight the central 50% of the distribution, while light colors correspond to the boxplot whiskers. (d-f) A random sample of 200 peaks (for visualization reason not all peaks are plotted), with colors highlighting the cluster membership. In both images, peaks are registered using as landmark the location of their maximum height. Peak shapes in the different clusters look very similar to the ones obtained in Replicate 1, shown in Fig. 5.

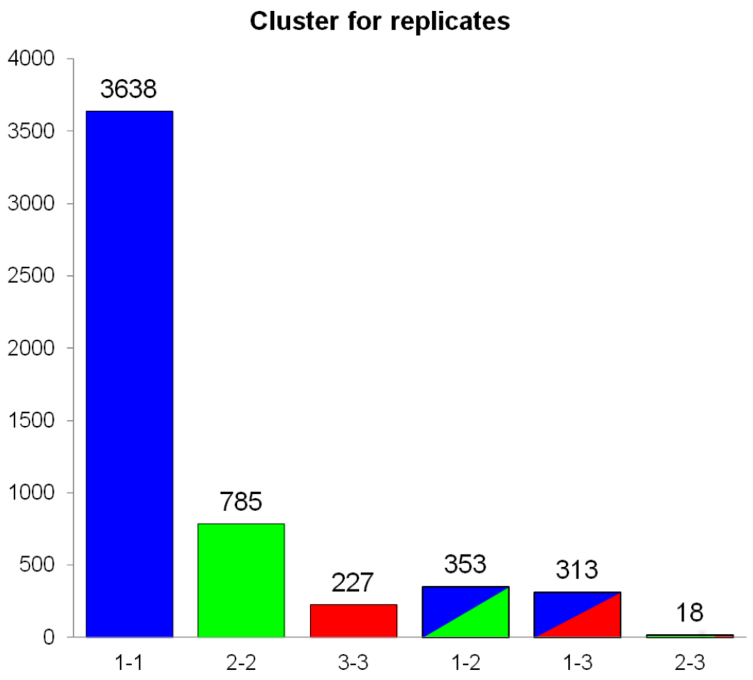

Figure S7. Robustness analysis results. Correspondences between cluster memberships of the 5534 peaks called in both replicates for K562 cells. The first three bars represents the 4650 peak pairs (~87%) that are classified in the same way in the two replicates, while the latest three bars correspond to the 684 misclassified pairs (~13%).

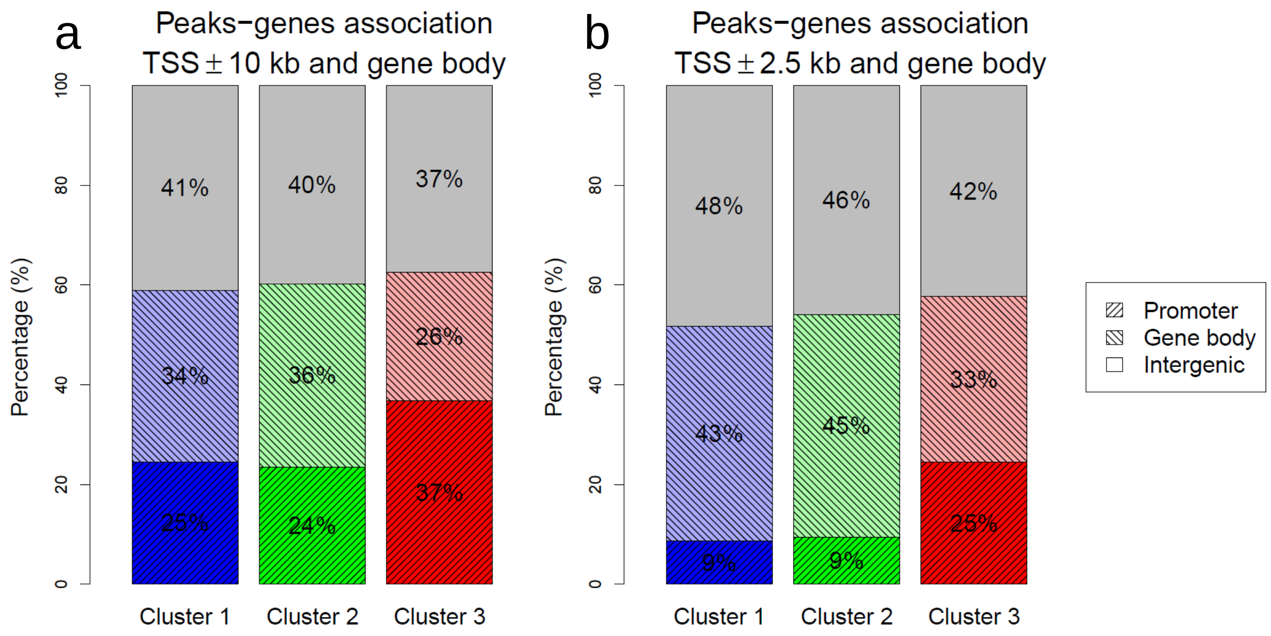

Figure S8. Peaks-genes association. Association between GATA-1 peaks and genes for the three clusters in K562 cells. Gray areas show the intergenic peaks, peaks found in promoter regions are in dark colors, and peaks located in a known gene body are in light colors. a) Association between peaks and genes when we define the promoter regions as ≤10 kb from the transcription start site. b) Association between peaks and genes when we define the promoter regions as ≤2.5 kb from the transcription start site. We observe that in both cases Cluster 3 is more associated to promoters than the other clusters (the p-values of the tests with alternative hypotheses that this proportion is greater than the one for Cluster 1 and 2 are 0 in both cases).

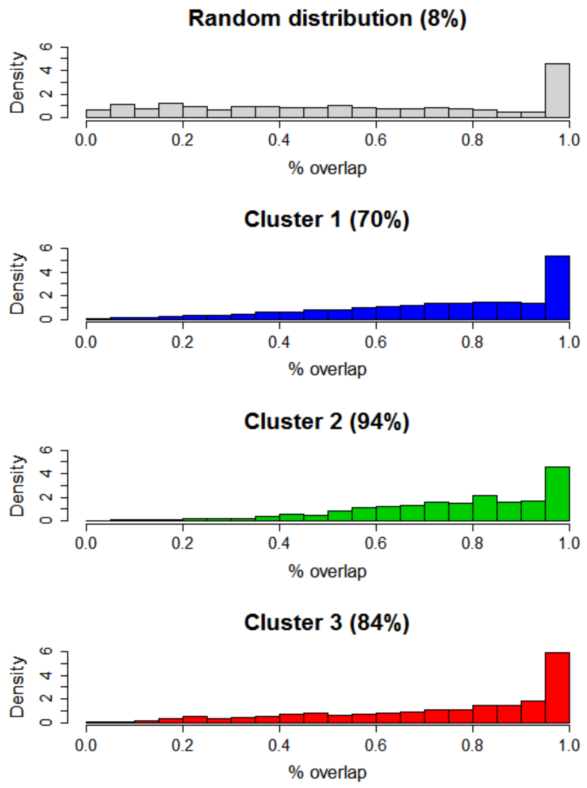

Figure S9. Overlap with open chromatin regions. Distribution of the percentage of intersection of the peak in each cluster of Replicate 1 for K562 cells with open chromatin regions. Only peaks that overlap OCRs are considered in these histograms.

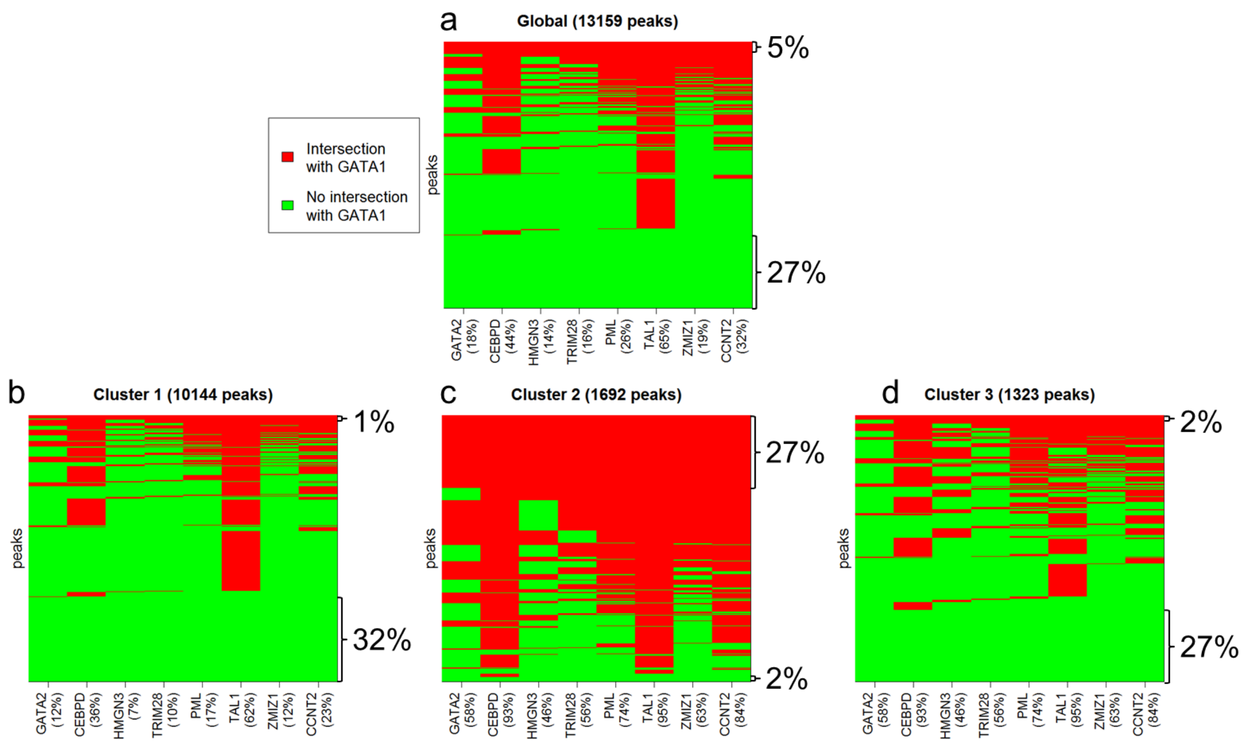

Figure S10. Combinatorial interaction analysis. Combinatorial interaction analysis on the eight transcription factors selected with random forests (see Table 2), in Replicate 1 for K562 cells. Here we consider an overlap when GATA-1 peak intersects all ChIP-Seq replicates for the regulatory element of interest. (a) Results considering all the peaks simultaneously. (b-d) Cluster specific results. In all plots, each row represents the overlap of a GATA-1 peak with the eight protein considered. The red color means that there is an intersection, while green stands for the absence of the protein in the correspondent GATA-1 region. The proportion of GATA-1 peaks that intersect the different proteins is indicated in brackets near the protein name.

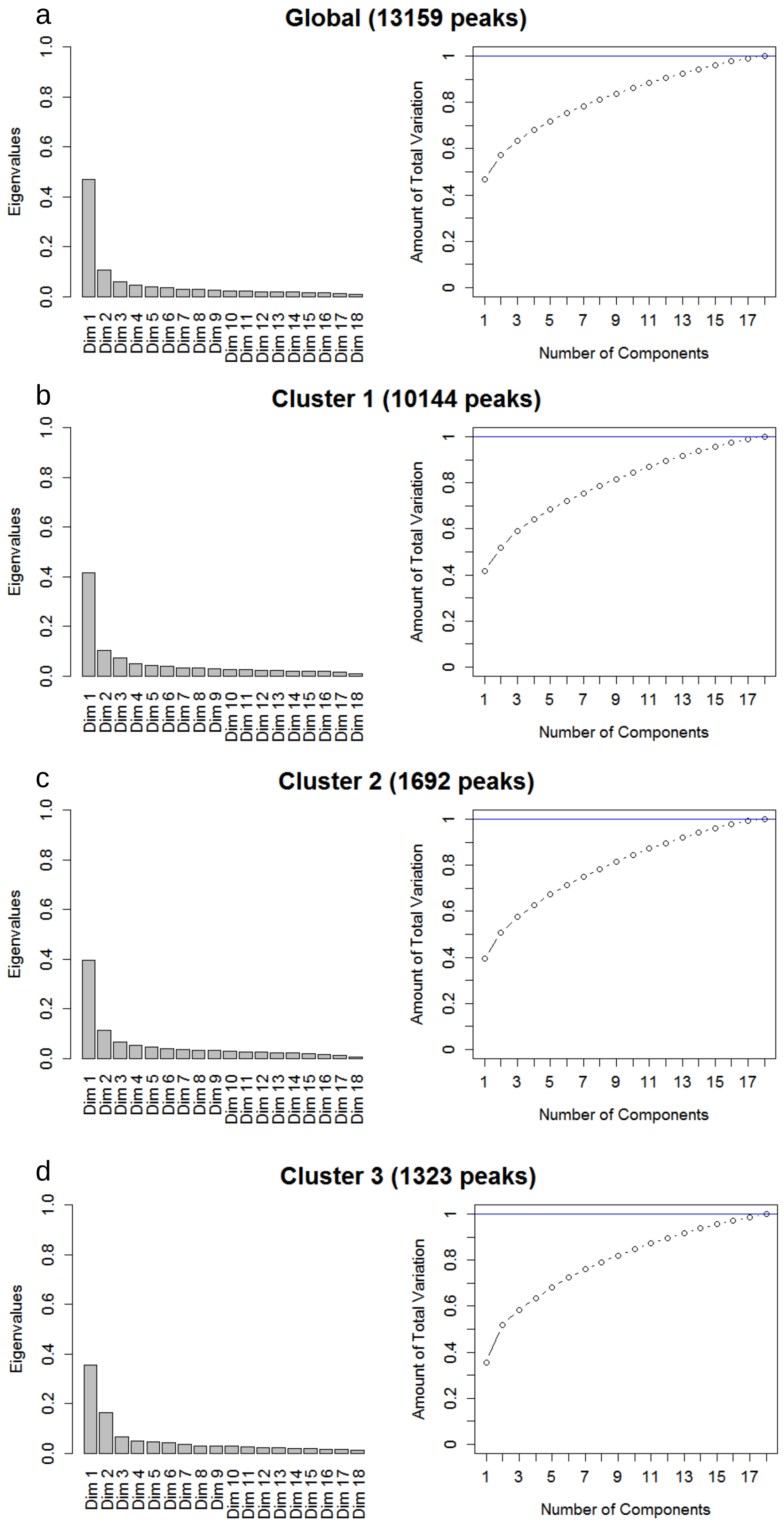

Figure S11. Multiple Correspondence Analysis. Eigenvalues and plot of the amount of total variation explained by an increasing number of principal components, in Replicate 1 for K562 cells.

(a) Multiple Correspondence Analysis results considering all GATA-1 peaks simultaneously. (b-d) The three different clusters are analyzed separately. In all cases we choose to use only the first two components.

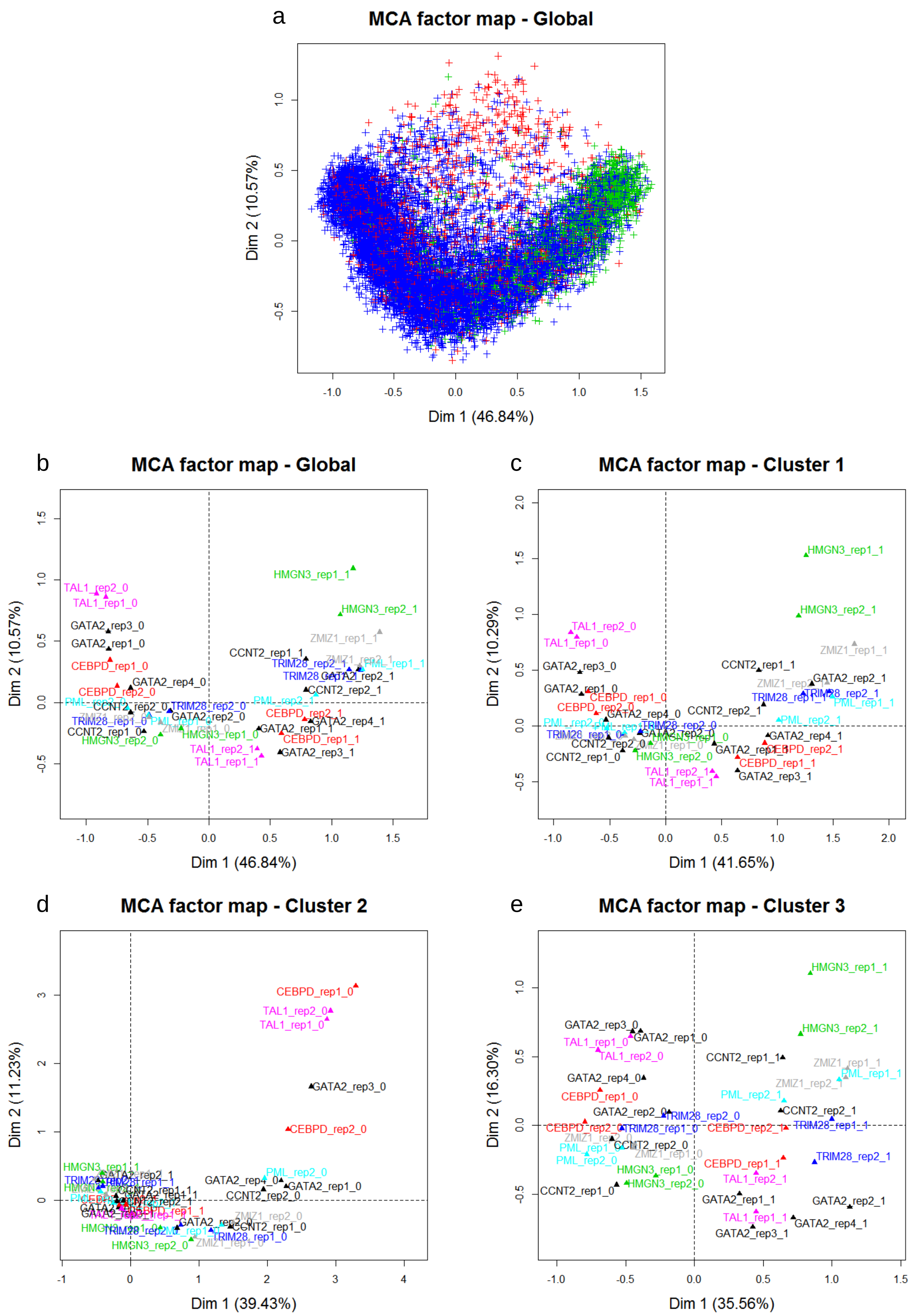
a
MCA factor map - Global
Dim 1 (46.84%)
Dim 2 (10.57%)
b
MCA factor map - Global
Dim 1 (46.84%)
Dim 2 (10.57%)
c
MCA factor map - Cluster 1
Dim 1 (41.65%)
Dim 2 (10.29%)
d
MCA factor map - Cluster 2
Dim 1 (39.43%)
Dim 2 (11.23%)
e
MCA factor map - Cluster 3
Dim 1 (35.56%)
Dim 2 (16.30%)

Figure S12. Multiple Correspondence Analysis factor maps. (a) Representation of the 13159 GATA-1 peaks (in Replicate 1 for K562 cells) in the space of the first two principal coordinates, with colors indicating cluster memberships. (b-e) Factor maps that show the different levels (1 means that there is an overlap, while 0 stands for no intersections) of each ChIP-Seq replicate selected with random forest analysis (see Table 2), in the same space induced by the first two principal coordinates, considering all peaks and the three clusters separately. Here colors indicate the eight transcription factors.

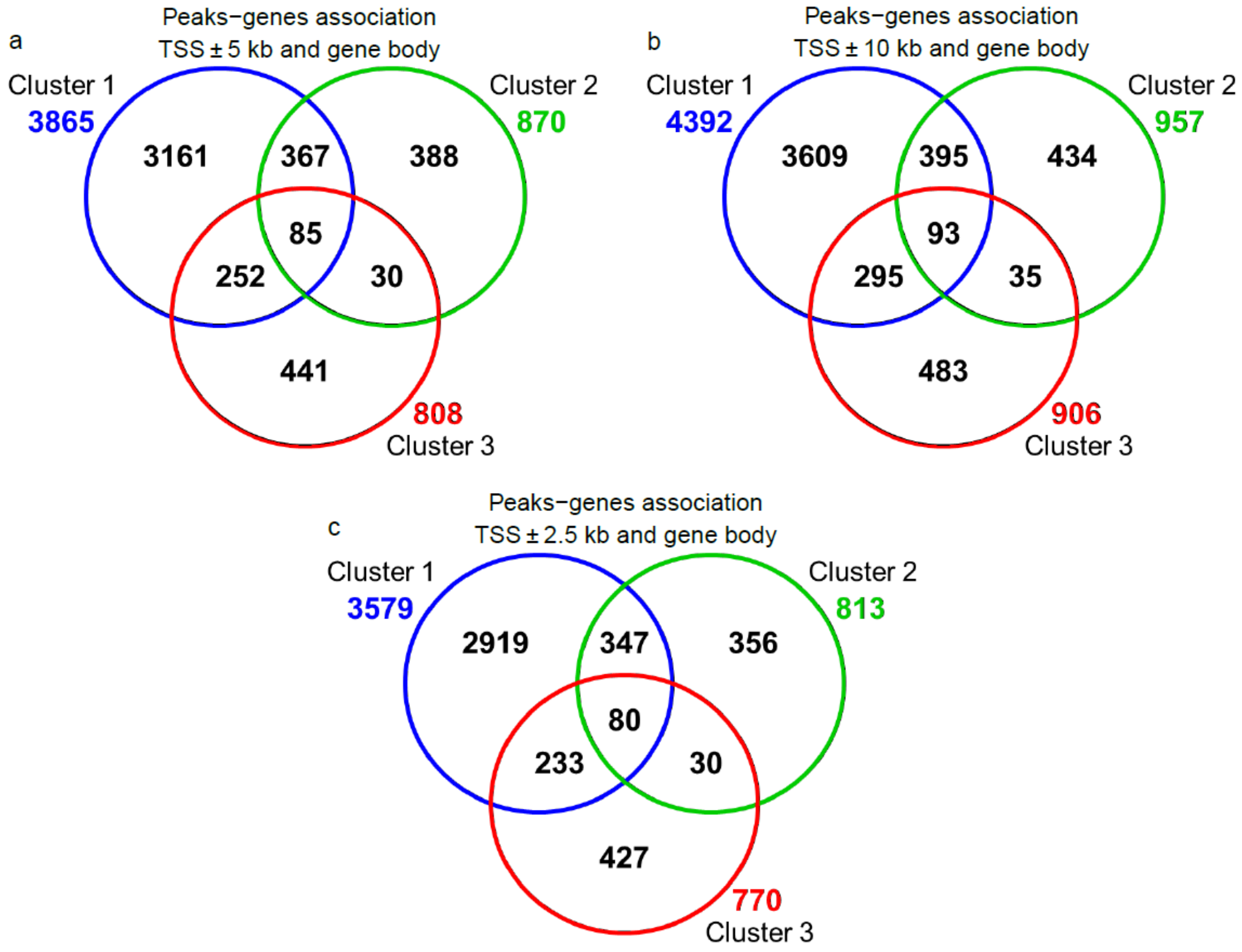

Figure S13. Association between peaks and genes. Venn diagrams of the known genes associated to GATA-1 clusters. (a) A gene is assigned to a peak if the peak is located less than 5 kb from the transcription start site, or in the gene body. (b) A gene is assigned to a peak if the peak is located less than 10 kb from the transcription start site, or in the gene body (less restrictive rule). (c) A gene is assigned to a peak if the peak is located less than 2.5 kb from the transcription start site, or in the gene body (more restrictive rule).

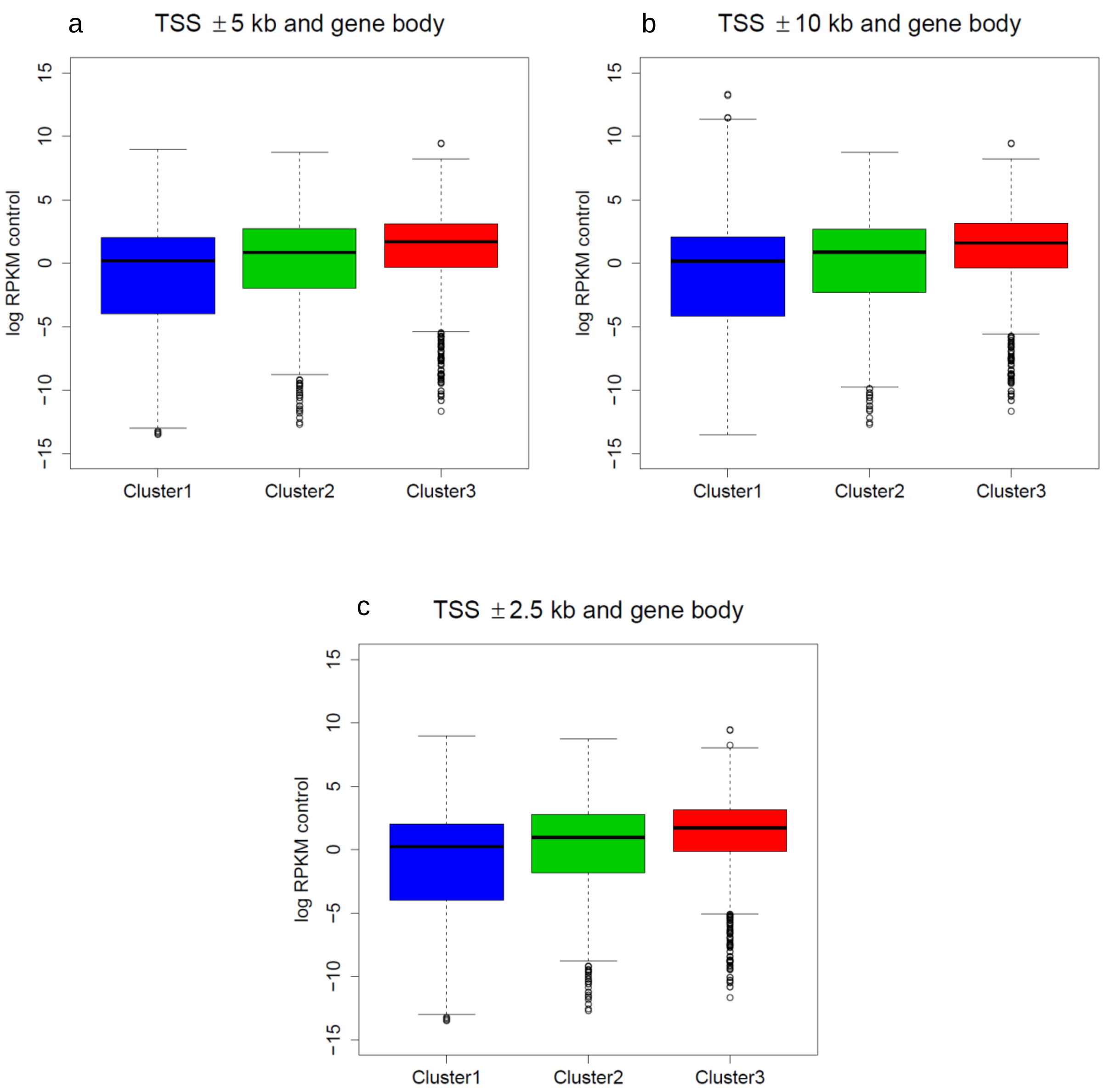

Figure S14. Base expression level. Boxplots of the base expression level (RNA-seq without any treatment) in Reads Per Kilobase per Million of the genes associated to the different cluster for K562 cells. (a) The association clusters-genes is done considering as promoter the region ≤5 kb from the TSS. (b) The association clusters-genes is done considering as promoter the region ≤10 kb from the TSS. (c) The association clusters-genes is done considering as promoter the region ≤2.5 kb from the TSS.

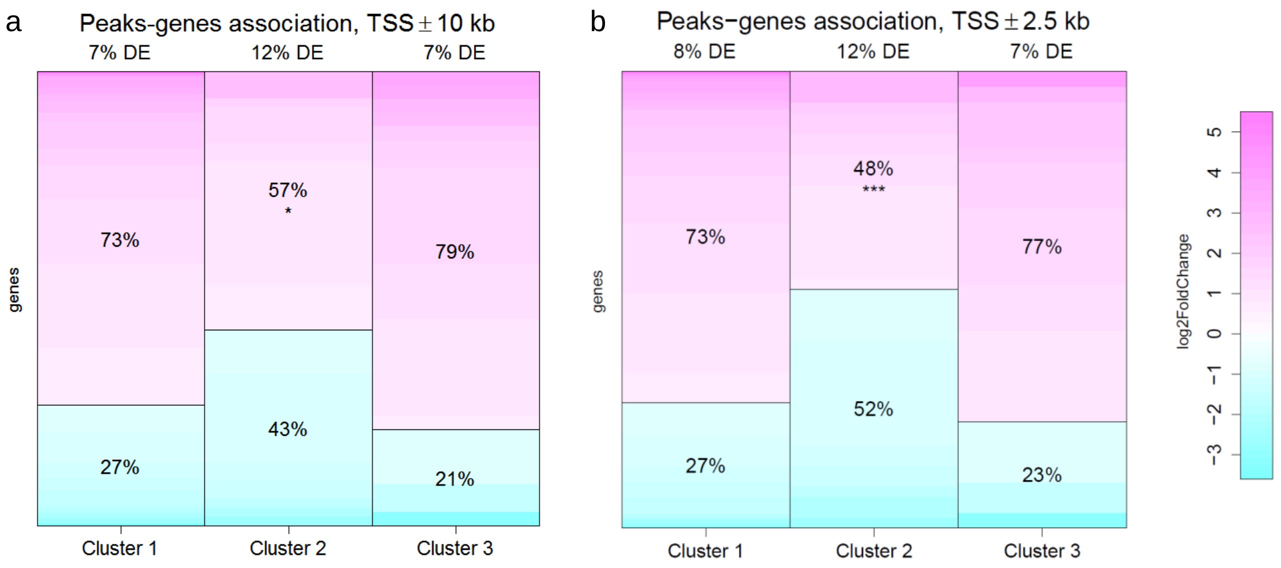

Figure S15. Gene expression analysis. Expression level fold change for differential expressed genes after knockdown of GATA-1 in K562 cells. The proportion of differential expressed genes in the different clusters is shown above each bar, where purple indicates overexpression, while cyan displays underexpressed genes. Only the known genes that are univocally associated to a single cluster are shown. Bonferroni corrected p-values of Fisher's exact test on the percentage of overexpressed genes (as explained in Method) are shown with asterisks (with codes: <0.001 '***', <0.01 '**', <0.05 '*'). The association between peaks and genes is done considering as promoter the region a) ≤10 kb from the TSS (less restrictive rule) and b) ≤2.5 kb from the TSS (more restrictive rule).

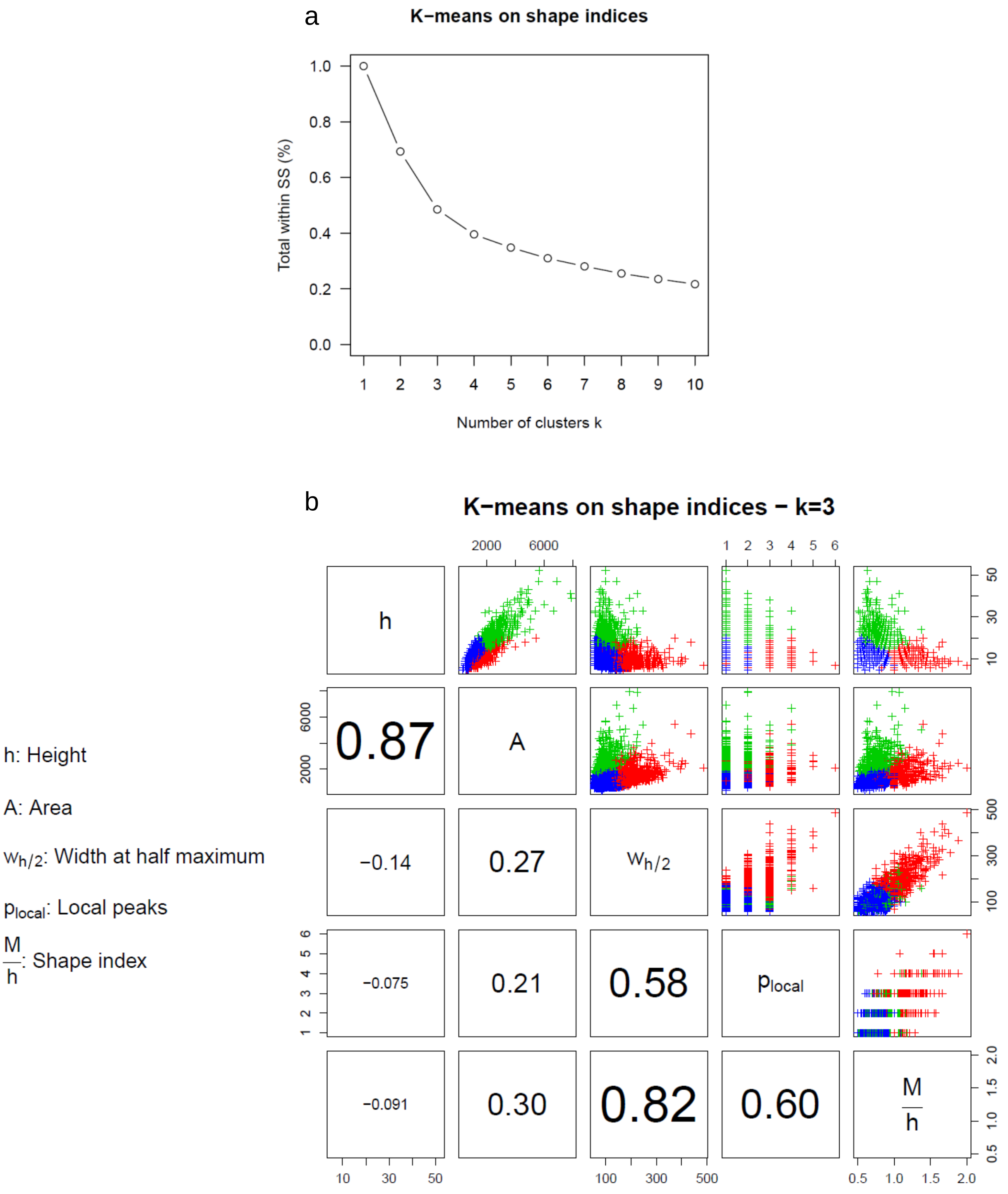

Figure S16. K-means results in megakaryocytes. Results of *k*-mean algorithm with Euclidean distance on the standardized shape indices, in megakaryocytes. (a) The total within-clusters sum of squares plot suggests that *k*=3 is the correct number of clusters. (b) Scatterplot of the five indices, with elements colored according the cluster they belong to after choosing *k*=3 in the *k*-mean results. The lower panels show the correlations between the different indices, considering all the peaks at the same time.

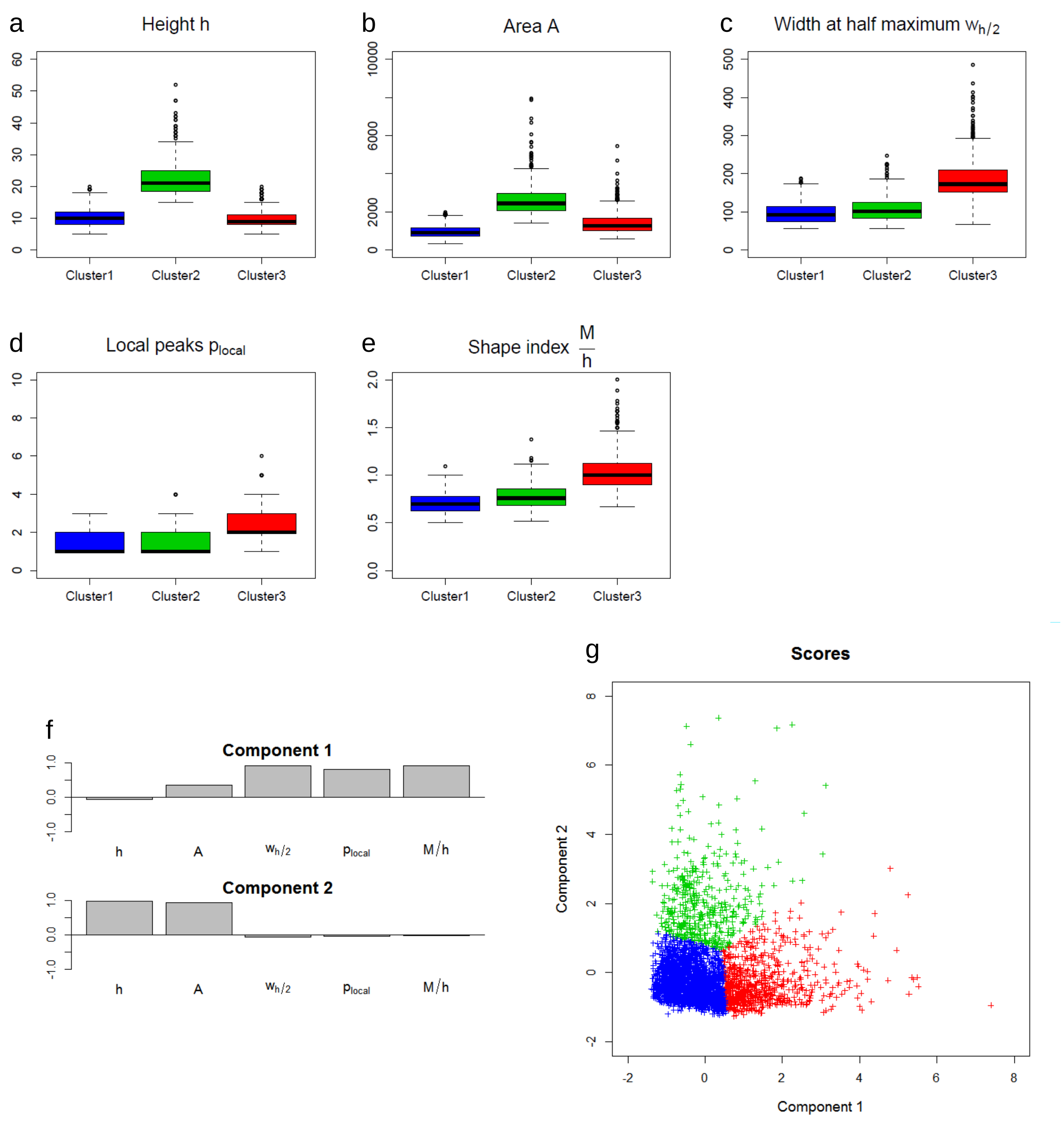

Figure S17. Distribution of shape indices in the three clusters in megakaryocytes. Results of *k*-mean algorithm with Euclidean distance on the standardized shape indices, in megakaryocytes. (a-e) Shape indices boxplots in the three clusters. (f) The first two components obtained with independent component analysis, in term of the initial shape indices. (g) Scatterplot of the data in the plane defined by the first two independent components, with each point representing a peak colored according to the cluster it belongs to.

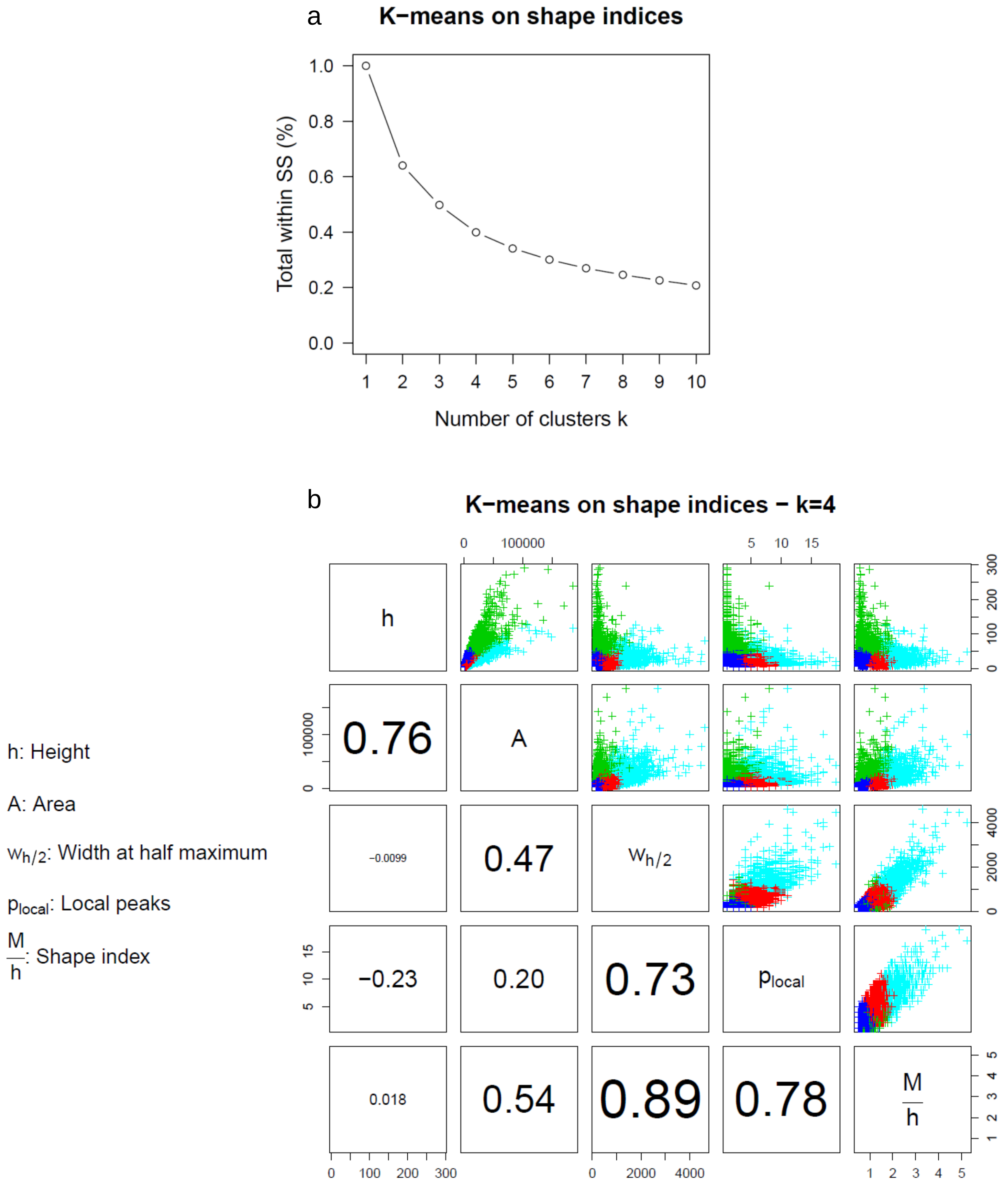

Figure S18. K-means results for CCNT2 in K562. Results of *k*-mean algorithm with Euclidean distance on the standardized shape indices, for CCNT2 in K562. (a) The total within-clusters sum of squares plot that suggests *k*=4 is the correct number of clusters. (b) Scatterplot of the five indices, with elements colored according the cluster they belong to after choosing *k*=4 in the *k*-mean results. The lower panels show the correlations between the different indices, considering all the peaks at the same time.

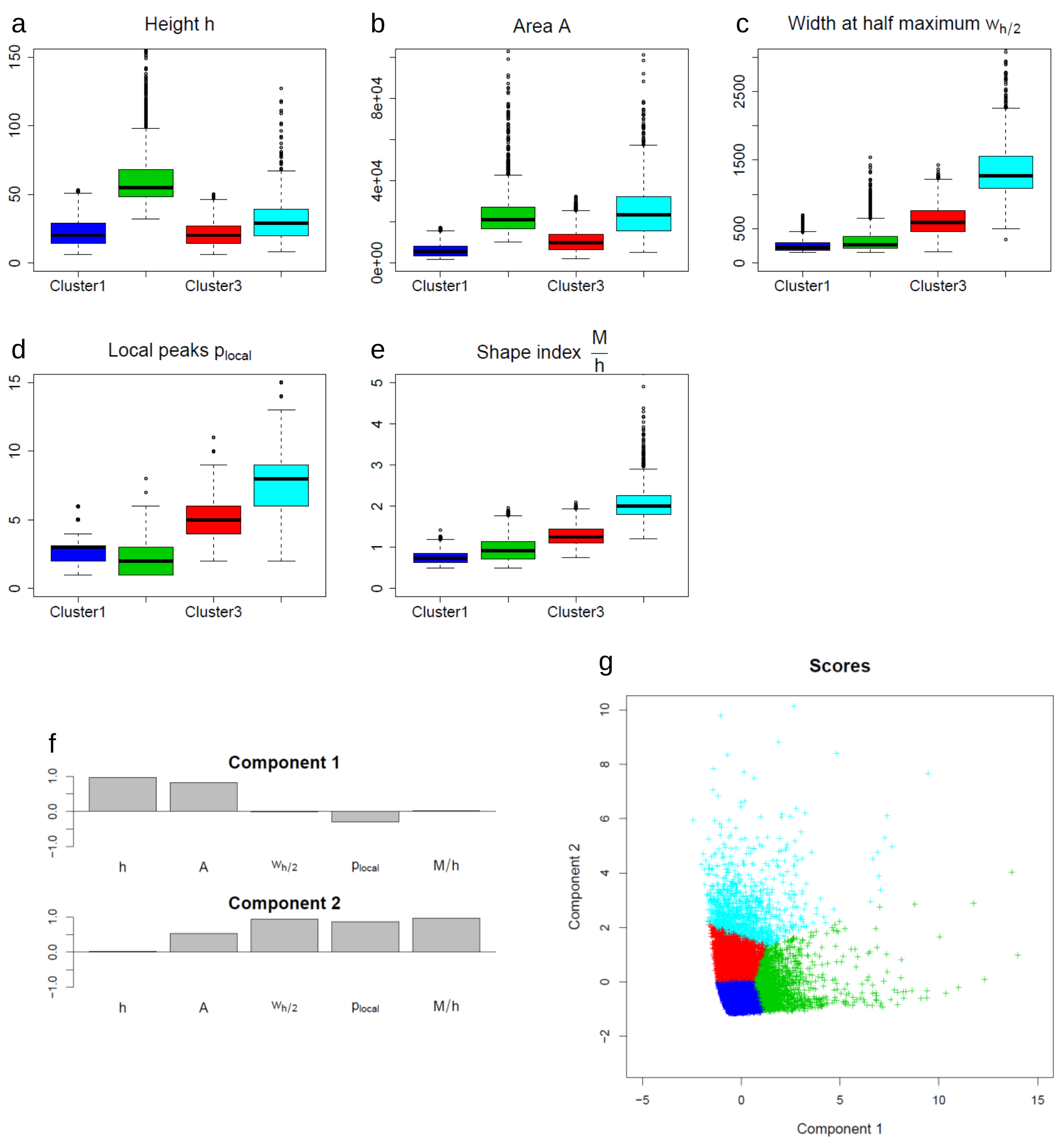

Figure S19. Shape indices distribution in the four clusters for CCNT2 in K562. Results of *k*-mean algorithm with Euclidean distance on the standardized shape indices, for CCNT2 in K562. (a-e) Shape indices boxplots in the four clusters. (f) The first two components obtained with independent component analysis, in term of the initial shape indices. (g) Scatterplot of the data in the plane defined by the first two independent components, with each point representing a peak and colored according to the cluster it belongs to.

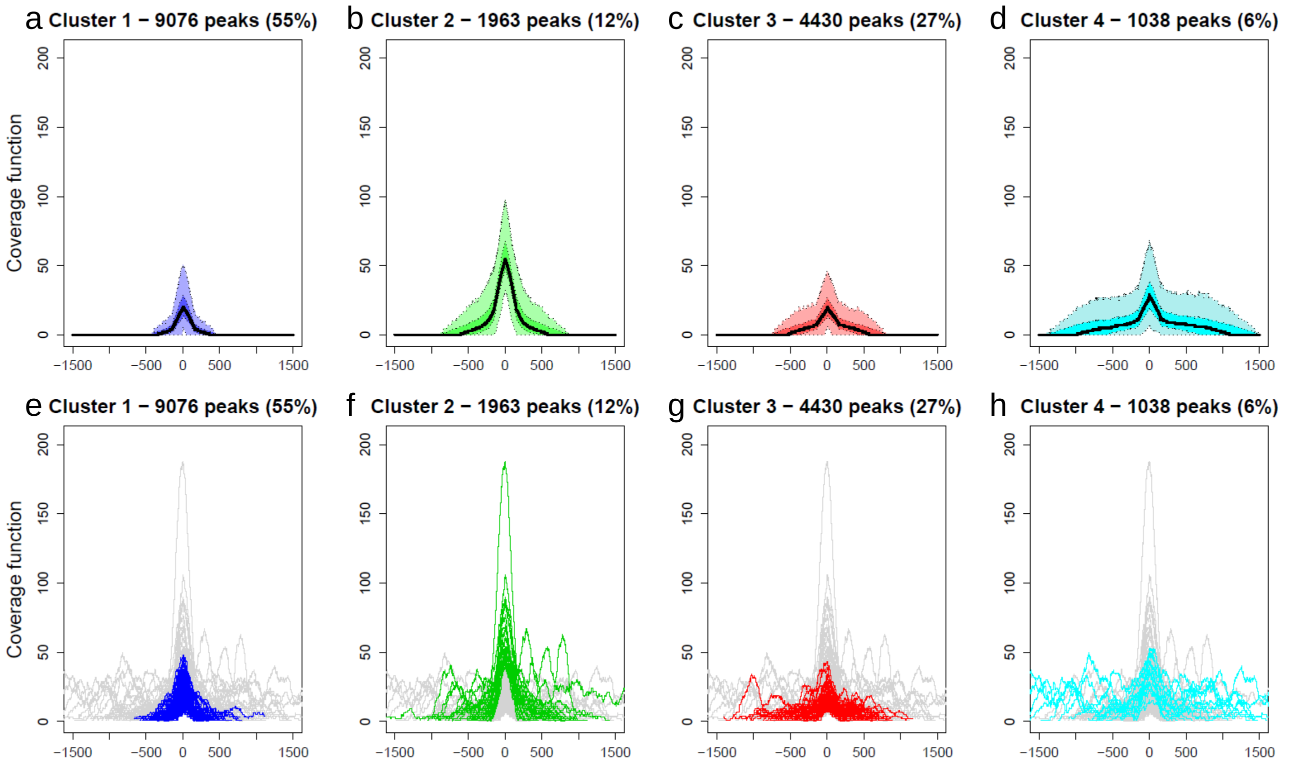

Figure S20. The three clusters obtained on shape indices for CCNT2 in K562. Results of *k*-mean algorithm with Euclidean distance on the standardized shape indices, for CCNT2 in K562 cells. (a-d) Pointwise boxplots of the coverage function in the four clusters. For each abscissa, black indicates the median value, dark colors highlight the central 50% of the distribution, while light colors correspond to the boxplot whiskers. (e-h) A random sample of 200 peaks (for visualization reason not all peaks are plotted), with colors highlighting the cluster membership. In both images, peaks are registered using as landmark the location of their maximum height.

			Filtered reads	Read length	Estimated fragment length	Filtered peaks
**K562**	**GATA-1 (sc-266)**	**Replicate 1**	16.5 Mil	32 bp	112 bp	13159
		**Replicate 2**	17.1 Mil	32 bp	112 bp	5509
	**GATA-1**		9.5 Mil	32 bp	65 bp	5012
**PBDE**	**GATA-1**		5.9 Mil	32 bp	279 bp	14173

Table S1. ChIP-Seq parameters. Relevant parameters obtained from the pre-processing on: a) the two ChIP-seq replicates for the transcription factor GATA-1 in K562 cells analyzed in section “GATA-1 in K562 cells” and plotted in Fig. 1a (rows 1 and 2 of the table); b) a ChIP-seq for the transcription factor GATA-1 in K562 cells, done with a different antibody and plotted in Fig. 1b-c (row 3 of the table); a ChIP-seq for the transcription factor GATA-1 in peripheral blood-derived erythroblasts, plotted in Fig. 1c (row 4 of the table).

GO Biological Process	Hypergeometric FDR p-value		
	Cluster 1	Cluster 2	Cluster 3
Erythrocyte differentiation	0.66	**1e-3**	0.10
Myeloid cell differentiation	0.10	**8e-4**	0.46
Erythrocyte homeostasis	0.65	**5e-3**	0.12

Table S2. Typical GATA-1 Gene Ontology terms. Results of enrichment analysis for the typical GATA-1 GO terms in the different clusters (experiments on K562 cells). Significant p-values (FDR<0.05) are reported in bold.

	GO Biological Process	Binomial FDR p-value	Hypergeometric FDR p-value
Cluster 1	Actin cytoskeleton reorganization	4e-13	7e-3
	Regulation of tyrosine phosphorylation of STAT protein	4e-12	1e-2
	Regulation of JAK-STAT cascade	4e-12	4e-2
	Regulation of protein complex disassembly	5e-9	2e-2
	Positive regulation of tyrosine phosphorylation of STAT protein	1e-7	4e-2
	Mitochondrial membrane organization	3e-7	4e-2
	Parasympathetic nervous system development	1e-6	3e-2
	Positive regulation of transforming growth factor beta receptor signaling pathway	8e-5	3e-2
	L-amino acid transport	2e-4	2e-2
	Pyramidine-containing compound transmembrane transport	3e-4	5e-2
Cluster 2	Erythrocyte differentiation	7e-8	1e-3
	Myeloid cell differentiation	7e-7	8e-4
	Erythrocyte homeostasis	1e-6	5e-3
	Homeostasis of number of cells	2e-4	7e-3
	Positive regulation of tyrosine phosphorylation of Stat3 protein	8e-3	5e-2
	Cellular response to cAMP	4e-2	2e-2
Cluster 3	Microspike assembly	4e-2	2e-2

Table S3. Gene Ontology results. Results of GO Biological Process enrichment analysis in the different clusters (experiments on K562 cells). Only terms that are significant (FDR<0.05) by both the binomial test over genomic regions and the hypergeometric test over genes are displayed. The complete table of significant by binomial test GO terms is in Additional file 3.

	Motif found	Known or similar motifs	E-value
**Cluster 1**	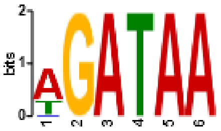	Gata4<br>GATA2<br>Gata6 primary	4e-1524
	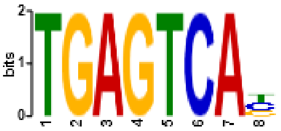	Bach1::Mafk<br>NFE2::MAF<br>HUND	5e-55
	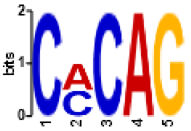	Tcf3<br>Zic2 secondary	6e-52
	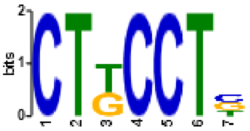	EHF<br>Erg<br>Spi1	4e-44
	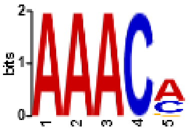	FOXD1<br>FOXP2<br>FOXO3	8e-33
	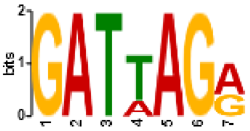	Tcf1 secondary<br>GATA3<br>DUX4	2e-26
	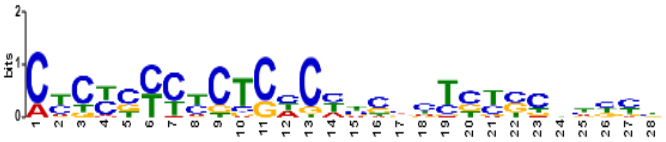	ZNF263<br>Zfp281 primary<br>SP2	2e-24
	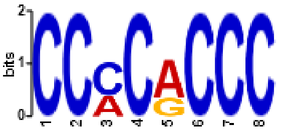	Klf4<br>KLF5<br>Klf1	2e-23
	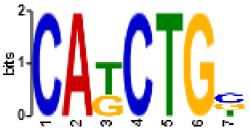	Atoh1<br>TAL1::TCF3<br>Tcfe2a secondary	1e-17
	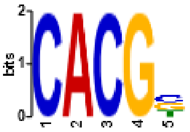	Pax2<br>Arnt::Ahr<br>HIF1A::ARNT	7e-17
	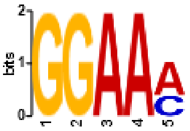	Bcl6<br>Ehf secondary<br>NFATC2	5e-9

	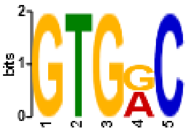	USF2<br>Max secondary<br>NR4A2	7e-6
	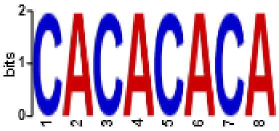	Sox7 secondary<br>Zscan4 secondary<br>Klf1	2e-4
	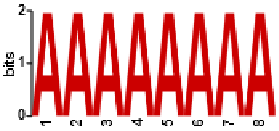	Srf secondary<br>Elf3 secondary<br>Tcfap2e secondary	2e-3
	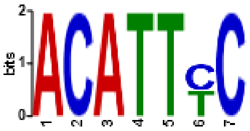	TEAD1	4e-3
	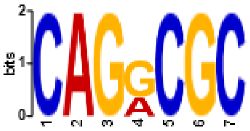	NRF1<br>Zfp161 primary<br>Myf6 secondary	1e-3
	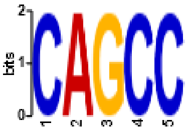		2e-2
	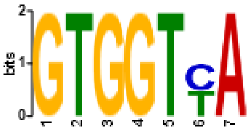	Bapx1 2343.1<br>Nkx2-2 2823.1<br>Nkx3-1 primary	2e-2
	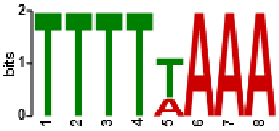	Srf secondary	4e-2
**Cluster 2**	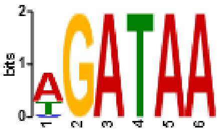	Gata4<br>GATA2<br>Gata6 primary	4e-265
	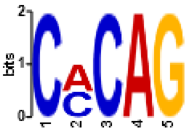	Tcf3<br>Zic2 secondary	2e-15
	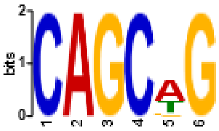	Zic1 secondary<br>Zic2 secondary<br>Zic3 secondary	1e-9

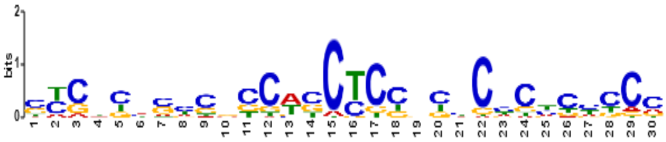	ZNF263<br>Zfp281 primary<br>Zfp740 primary	2e-8
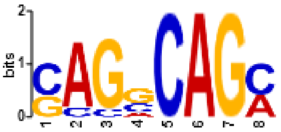		3e-5
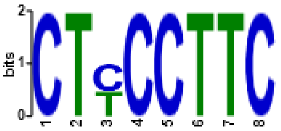	EWSR1-FLI1	5e-4
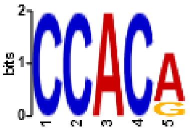	RUNX1<br>Klf1<br>RUNX2	6e-4
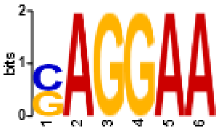	EHF<br>Erg<br>SPIB	2e-2

Cluster 3

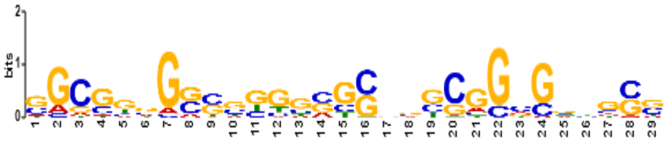	EGR1<br>Zfp281 primary<br>SP2	9e-26
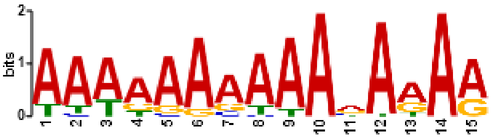	Zfp105 primary<br>FOXP1<br>Mtf1 secondary	2e-25
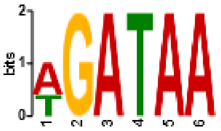	Gata6 primary<br>GATA2<br>Gata4	7e-21
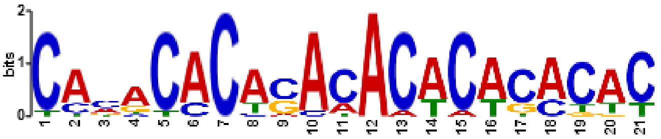	EGR2<br>RREB1<br>Gm397 secondary	2e-9
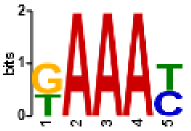	Stat5a::Stat5b	3e-6
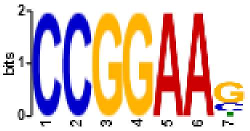	GABPA<br>ELK4<br>Gabpa primary	1e-5

	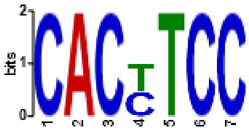	GABPA<br>ELF1<br>Spi1	1e-3
	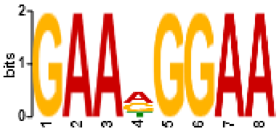		5e-2

Table S4. Motif analysis. MEME-ChIP motif analysis results on Replicate 1 for K562 cells, done by using all the peaks in each cluster.

	Peaks in OCRs
**Random**	776 (8%)
**Global**	9754 (74%)
**Cluster 1**	7055 (70%)
**Cluster 2**	1590 (94%)
**Cluster 3**	1109 (84%)

Table S5. Overlap with open chromatin regions. Number of peaks (percentage) that overlap open chromatin regions. The three clusters are considered, as well as the global and the random cases, in Replicate 1 for K562 cells.
